# Loss of the matrix metalloproteinase-10 causes premature features of aging in satellite cells

**DOI:** 10.3389/fcell.2023.1128534

**Published:** 2023-05-09

**Authors:** Miriam Bobadilla Muñoz, Josune Orbe, Gloria Abizanda, Florencio J. D. Machado, Amaia Vilas, Asier Ullate-Agote, Leire Extramiana, Arantxa Baraibar Churio, Xabier L. Aranguren, Gloria Cantero, Neira Sáinz Amillo, José Antonio Rodríguez, Luis Ramos García, Juan Pablo Romero Riojas, Ainara Vallejo-Illarramendi, Carmen Paradas, Adolfo López de Munain, José Antonio Páramo, Felipe Prósper, Ana Pérez-Ruiz

**Affiliations:** ^1^ Regenerative Medicine Program, Center for Applied Medical Research (CIMA) Universidad de Navarra, CIBERONC, Madrid, Spain; ^2^ Instituto de Investigación Sanitaria de Navarra (IdiSNA), Pamplona, Spain; ^3^ Laboratory of Atherothrombosis, Program of Cardiovascular Diseases, CIMA Universidad de Navarra, Pamplona, Spain; ^4^ Redes de Investigación Cooperativa Orientadas a Resultados en Salud (RICORS)-Ictus, Instituto de Salud Carlos III, Madrid, Spain; ^5^ Instituto de Biomedicina de Sevilla (IBiS), Hospital Universitario Virgen del Rocío/CSIC/Universidad de Sevilla, Neuromuscular Disorders Unit, Sevilla, Spain; ^6^ Centro de Investigación Biomédica en Red sobre Enfermedades Neurodegenerativas (CIBERNED), Madrid, Spain; ^7^ Centre for Nutrition Research, Universidad de Navarra, Pamplona, Spain; ^8^ Centro de Investigación en Red de Enfermedades Cardiovasculares (CIBERCV), Instituto de Salud Carlos III, Madrid, Spain; ^9^ Radiology Department, Clínica Universidad de Navarra, Pamplona, Spain; ^10^ Radiology Department, Osakidetza Basque Health Service, Donostialdea Integrated Health Organisation, San Sebastian, Spain; ^11^ CIBERNED-Biodonostia, Neurosciences Area, Group of Neuromuscular Diseases, San Sebastian, Spain; ^12^ Neurology Department, Osakidetza Basque Health Service, Donostialdea Integrated Health Organisation, San Sebastian, Spain; ^13^ Hematology Service, Clínica Universidad de Navarra, Pamplona, Spain

**Keywords:** MMP-10, satellite cell, muscle repair, DNA damage, aging, senescence, ECM

## Abstract

Aged muscles accumulate satellite cells with a striking decline response to damage. Although intrinsic defects in satellite cells themselves are the major contributors to aging-associated stem cell dysfunction, increasing evidence suggests that changes in the muscle-stem cell local microenvironment also contribute to aging. Here, we demonstrate that loss of the matrix metalloproteinase-10 (MMP-10) in young mice alters the composition of the muscle extracellular matrix (ECM), and specifically disrupts the extracellular matrix of the satellite cell niche. This situation causes premature features of aging in the satellite cells, contributing to their functional decline and a predisposition to enter senescence under proliferative pressure. Similarly, reduction of MMP-10 levels in young satellite cells from wild type animals induces a senescence response, while addition of the protease delays this program. Significantly, the effect of MMP-10 on satellite cell aging can be extended to another context of muscle wasting, muscular dystrophy. Systemic treatment of *mdx* dystrophic mice with MMP-10 prevents the muscle deterioration phenotype and reduces cellular damage in the satellite cells, which are normally under replicative pressure. Most importantly, MMP-10 conserves its protective effect in the satellite cell-derived myoblasts isolated from a Duchenne muscular dystrophy patient by decreasing the accumulation of damaged DNA. Hence, MMP-10 provides a previously unrecognized therapeutic opportunity to delay satellite cell aging and overcome satellite cell dysfunction in dystrophic muscles.

## Introduction

Satellite cells are the adult stem cells of skeletal muscle, since they satisfy the classical definition of tissue-specific adult stem cells. Satellite cells are normally quiescent, but they have the ability to activate, proliferate and differentiate upon injury, restoring or replacing damaged myofibers, while replenishing the satellite cell compartment to ensure muscle responses in future injuries (Chargé and Rudnicki, 2004; Collins and Partridge, 2005). To appropriately fulfil these functions, satellite cells must maintain a dynamic balance between their different cell states through a tight regulation, as any imbalance may result in progressive tissue failure, as occurs in aging. Aged satellite cell function is dysregulated by intrinsic and extrinsic mechanisms. Intrinsic mechanisms include specific transcription factors expressed by the cells, such as the p38 MAPK, JAK-STAT3 and p16Ink4a signaling pathways, which are associated with cellular stress. Extrinsic mechanisms involve signals provided by the local microenvironment (niche) such as growth factors, contact with other cells and the extracellular matrix (ECM). Intrinsic defects offer the greatest insights into the regulatory events underlying satellite cell dysfunction and muscle loss of homeostasis in aging. Nevertheless, extrinsic cues provided by the niche may be responsible for satellite cells acquiring intrinsic defects (Brack and Muñoz-Cánoves, 2016; Hwang and Brack, 2018; Fuchs and Blau, 2020; Sousa-Victor et al., 2020; Hong et al., 2022; Brunet et al., 2023).

In skeletal muscle, satellite cells reside in the niche between the myofiber plasma membrane and the basal lamina (Schüler et al., 2022), a network of ECM components mainly composed of collagens, glycoproteins and proteoglycans. As a key component of the satellite cell niche, the ECM is not just an inert scaffold to maintain tissue and organ structure but, rather can profoundly influence stem cell fate choices (Loreti and Sacco, 2022a; Schüler et al., 2022). Accordingly, any change in the satellite cell niche ECM structure and composition may represent one mechanism by which stem cell compartments age. Therefore, ECM modifiers such as metalloproteinases (MMPs) (Fanjul-Fernández et al., 2010) may participate in this process. Not surprisingly, dysregulated protease function is a common characteristic of aging and premature aging disorders (Freitas-Rodríguez et al., 2017) and a deficiency of the membrane type 1-MMP membrane-anchored protease causes cell senescence, contributing to aging (Gutiérrez‐Fernández et al., 2015). MMP-10, which belongs to the stromelysin family of MMPs and it is expressed by different cell populations within skeletal muscle (Bobadilla et al., 2014), can cleave diverse ECM components such as collagens III, IV and V, aggrecan, fibronectin, laminin, proteoglycans, versican, perlecan, decorin, entactin and tenascin, among others (Fanjul-Fernández et al., 2010). This proteolytic function can explain why MMP-10 loss in young mice modifies some muscle ECM components and satellite cells are unable to maintain an appropriate repair response after injury, while MMP-10 intramuscular injection restores muscular regeneration (Bobadilla et al., 2014). Therefore, because aging changes the muscle ECM (Kragstrup et al., 2011; Freitas-Rodríguez et al., 2016; Fuchs and Blau, 2020), we hypothesized that MMP-10 deficiency would cause satellite cell aging through abnormal proteolytic processing of the muscle ECM, thus disrupting the niche ECM.

## Materials and methods

### Mice

Wild-type (WT, C57BL/6J) mice were purchased from ENVIGO, the MMP-10 knockout animals were provided by Dr. WC Parks (University of Washington, Seattle, United States) and B6Ros.Cg-Dmd*mdx*-5cv/J (*mdx*) mice were obtained from Jackson Laboratory. When required, animals were left to age up to 2 and 18 months old in our Animal housing facilities, according to current legislation. All animal procedures performed in this study were subjected to the European and Spanish legal regulations.

### Cell culture

Mice were killed by cervical dislocation and the hind limb muscles isolated and mechanically disaggregated and dissociated in Ham’s F10 media containing collagenase II 0.2% (Worthington)/CaCl_2_ (Sigma; 2.5 mM) at 37°C for 30 min followed by a digestion with collagenase D (Roche; 1.5 U/ml)/Dispase (Roche; 1 U/ml) at 37°C for 60 min. Cells were incubated in lysing buffer (BD Pharm Lyse) for 10 min on ice and re-suspended in phosphate-buffered saline (PBS) with 2.5% goat serum. PE-Cy7-conjugated anti-CD45 (Biolegend 103113/14), anti-CD31 (Biolegend 10241/8), anti-CD11b (Biolegend 101215/16) and anti-Sca-1 (Biolegend 108113/14) antibodies were used to exclude the lineage negative population. Alexa647-conjugated anti-CD34 (BD Pharmigen 560230) and PE-conjugated anti-α7-integrin (Ablab 53-0010-05; clone R2F2) antibodies were used for double positive staining of quiescent satellite cells. When required, linage negative (Lin^−^) cells were sorted (CD31^+^/CD11b^+^/CD45^+^). Fibro-adipogenic progenitors (FAPs) were isolated from muscle-associated cell populations by using FITC-conjugated anti-Sca-1 antibody (Biolegend). Cells were sorted using a FACS Aria II flow cytometer (BD). Satellite cells were cultured in mitogen-rich media (Dulbecco’s modified Eagle’s medium (DMEM) with 20% fetal bovine serum (Millipore), 10% horse serum (HS) (Gibco), 1% chick embryo extract (ICN Flow), 2 mM L-glutamine and 1% penicillin-streptomycin), in culture plates coated with 1 mg/mL Matrigel (Corning) at 37°C in 5% CO_2_. When required, satellite cells were added to Matrigel-treated dishes and cultured in mitogen-rich media for 3 days (myoblasts). Satellite cell-derived myoblasts were maintained for three additional days in 2% HS media to induce myoblast fusion into myotubes.

Single myofibers with their associated satellite cells were carefully isolated from the *extensor digitorum longus* (EDL) muscles of mice. EDL muscles were incubated in filter-sterilized 0.2% (wt/vol) type I collagenase (Sigma) at 37°C for 90 min. Following digestion, the muscle was transferred to plastic dishes containing 8 mL of DMEM using a wide-mouth, fire-polished-tip Pasteur pipette, which was previously treated rinsed in 10% horse serum in DMEM. Under a transilluminating stereo dissecting microscope, single muscle fibers were liberated by repeatedly triturating the muscle with a wide-mouth Pasteur pipette. The intact, viable muscle fibers were then separated from debris by transferring them in a fresh Petri dish. Basal media was removed and replaced by growth medium (DMEM with 10% horse serum, 0.5% chick embryo extract, 2 mM L-glutamine, and 1% penicillin-streptomycin) and fibers were incubated at 37°C and 5% CO_2_ for 72 h. When required, single EDL fibers were plated onto Matrigel, the fibers removed 2 days later, and satellite cells maintained in mitogen-rich media.

Human myoblasts from DMD patients and healthy people were cultured in Skeletal Muscle Cell Growth Medium (Promo-cell; C-23060) supplemented with 1% penicillin-streptomycin-amphotericin B. Human cells were expanded and cryopreserved. Experiments were performed in two or four different thawed cell aliquots. When required, cells were passaged to avoid cell fusion.

### siRNA transfection

Transfection of the satellite cells with *siRNAs* was performed in 6-well plates under proliferative conditions. Two consecutive transfections were performed, the second one was carried out 24 h after the first. mRNA levels of targeted genes were analyzed by qPCR after 24 h, while proteins were measured after 48 and/or 72 h by Western-blot. Cells were maintained in proliferation media avoiding myogenic differentiation. siRNA duplexes (*siMMP-10* or *siControl; Ambion*) were diluted in OptiMEM (Invitrogen) at 20 pmol/well and incubated with Lipofectamine 2,000 (Invitrogen) diluted in OptiMEM for 20 min at room temperature, according to the manufacturer’s instructions.

### MMP-10 treatment

For the *in vitro* studies, 2 nM of activated rhMMP-10 (MMP-10) were supplemented in growth medium and added daily to muscle cells. When required, cells were passaged to avoid cell fusion. For the *in vivo* studies, a single dose of MMP-10 (100 ng/mL) in Tween-saline buffer was injected into the tail vein of the *mdx* mice. Control-*mdx* mice received the same volume of Tween-saline buffer.

### Model of muscle injury

In order to induce muscle injury, young and old mice were anaesthetized by isoflurane inhalation and received intramuscular injections of 10 μL of *Notechis scutatus scutatus* notexin (Latoxan; 10 μg/mL) into the *tibialis anterior* (TA) muscles. A second round of injury was induced after 7 days, and mice were sacrificed by cervical dislocation 1 week later. In some animals, notexin was injected into the TA, quadriceps and gastrocnemius to isolate by FACS activated satellite cells, Lin^−^ and FAPs cells 3 days after injury.

### Viral production and infection

Viruses were produced by transfecting HEK293T packaging cells with pMSCV-IRES-eGFP constructs and a helper plasmid using Lipofectamine (Invitrogen) at 37°C. Suspension was collected 48 h later, aliquoted and frozen. Viral suspension was treated with polybrene (final concentration of 4 μg/mL) and added to the cells, which were incubated at 37°C. The next day, cells were washed in PBS, collected, counted and injected into the TA muscles of recipient mice.

### Cell transfer assays

Two days before cell delivery, the TA muscles of recipient mice received an episode of injury by Notexin injection. One day later, mice were anaesthetized with ketamine/xylazine (80/10 mg/kg), intraperitoneally, and their hind limb muscles were irradiated (18 Gy). 10.000 satellite cells from donor mice, infected with the *pMSCV-IRES-eGFP* virus, were transferred into the TA injured muscles of recipients. After 28 days, some recipients were sacrificed, while others received a second episode of damage and were sacrificed 3 weeks later. One day before cell delivery, recipients received Tacrolimus (0.4 mg/mL; Prograf, Astella Pharma S.A.) to minimize any immune reaction. This treatment was administered daily after transplantation. Anti-Asialo GM1 (1:200; Wako Pure Chemical Industries) was intraperitoneally injected every 10 days to avoid natural killer cell activity.

### Serum creatine kinase measurement

Animals were anaesthetized with isoflurane and blood was collected from the external maxillary vein using heparin-coated capillary tubes (Microvette ^®^ CD300, Sarsted). Serum was obtained by centrifugation at ×1,500 g for 10 min. Enzyme activity was measured by using a specific kit (Roche diagnosis) and was expressed in units per liter (U/L).

### Grip muscle strength

Grip strength was measured as tension force using a computerized force transducer (Grip Strength Meter, Bioseb), following the SOP DMD_M.2.2.001 protocol (Aartsma-Rus et al., 2008). Three blinded trials of three measurements per trial were carried out in each animal with a few minutes resting period between each. The average tension force related to total body weight was calculated for each group of mice.

### Immunostaining

Suspended myofibers and plated cells were fixed in 4% paraformaldehyde in PBS for 10 min, followed by several PBS rinses. After permeabilization with 0.5% Triton X-100/PBS, non-specific antibody binding was blocked using 20% goat serum/PBS. Skeletal muscles were frozen in isopentane cooled in liquid nitrogen, and serial 9-μm cryosections were collected at 100-μm intervals through the entire muscle. Sections were fixed in 10% formalin for 20 min and rinsed in PBS. When required, tissue sections were permeabilized with 0.1% Triton X-100/PBS, Tris-HCl, citrate buffer or with 0.2% Trypsin/CaCl_2_, and blocked.

The following primary antibodies were applied (4°C, overnight): mouse anti-myogenin, anti-Pax7, anti-eMyHC (Developmental Studies Hybridoma Bank, DSHB), anti-MyoD (DakoCytomation), anti-γH2AX (Cell Signalling), rat anti-Ki67 (eBioscience), anti-CD45 (Biolegend), anti-PDGFRα (Bioscience), rabbit anti-MyoD, anti-myogenin, anti-𝛽-dystroglican (Santa Cruz), anti-fibronectin, anti-collagen IV, anti-MMP-10 (Acris; abcam), anti-γH2AX, anti-HP1γ (Cell Signalling), anti-laminin (Sigma), and anti-GFP (Molecular probes). Primary antibodies were visualized with fluorochrome-conjugated secondary antibodies (goat/donkey anti-mouse-488/594/647 and/or goat/donkey anti-rabbit-488/594/647 and/or goat anti-rat-594/647; Molecular Probes) or with biotin-labelled secondary antibodies followed by TSA amplification kit before mounting in Faramount fluorescent mounting medium containing 4,6-diamidino-2-phenylindole (DAPI; 100 ng/mL; Molecular Probes).

Same immunostainings were carried out in at least three different animals/cells per condition, using 15–20 myofibers, two different tissue sections or 15-20 independent fields per sample.

### Senescence-associated *β*-galactosidase cytochemical staining

Satellite cells were cultured in growth media in 12-well plates and pushed to age by consecutive passages (every 2 days). Human cells were cultured for 8 days. Myogenic differentiation in mouse and human cells was avoided by culturing cells at a low density. Cells were fixed and the senescence-associated *β*-gal (SA-𝛽-gal) activity was determined using the Cellular Senescence Detection Kit (Biolabs, San Diego, CA, United States), according to the manufacturer´s instructions. Staining was performed for 24 h at 37°C, pH 6, and mounted in glycerol/PBS containing solution.

### Digital image acquisition and quantification

Immunostained myofibers, cells and muscle tissue sections were viewed using a Zeiss Axiophot epifluorescence microscope. When required, Z-stack images were captured (63X). The digital images thus obtained were processed using AxioVision and ImageJ software. When needed, images were composed and edited, and modifications were applied to the whole images using Photoshop CS6 (Adobe).

The distribution of the myofiber-associated satellite cells according to different myogenic phenotypes was determined by counting positive cells in at least 15–20 myofibers from at least three different animals per condition. Values were expressed as the percentage of total stained satellite cells.

In muscle tissue sections, collagen type IV, laminin, fibronectin and *β*-dystroglican images of Pax7^+^ satellite cells were taken by ZStacks projections (63X) at the same exposure time. The abundance of ECM surrounding the satellite cells was calculated using the Fiji/ImageJ program. To highlight protein disorganization in the niche ECM and identify contact disruption or abnormal accumulation with the satellite cells, ECM protein images were similarly modified. Normal ECM distribution was attributed to that continuously deposited covering the basal side of each Pax7^+^ satellite cell. Positive staining for IgG-Cy3, eMyHC and CD45 was expressed as the percentage of the stained area divided by the total area of muscle. Pax7^+^ satellite cells were counted in two whole muscle sections from each animal in at least three animals per group and related to the area of the muscle or counted in 100 regenerating myofibers. In cell delivery studies, the total number of GFP^+^ regenerating fibers and Pax7^+^ satellite cells were counted in any whole muscle sections of the recipients and compared to those found in control group (wild type satellite cells delivered into wild type recipients), which were considered as 100%. The total number of positive γHAX^+^ cells was related to the total area of the muscle (μm^2^) from at least three animals per condition, including two sections per animal. The HP1γ^+^ foci in the Pax7^+^ satellite cells were counted after acquiring ZStacks image projections (63X) at the same exposure time. ZStacks of Pax7^+^γHAX^+^ of satellite cells were acquired (63X), fixing shutter speed. In dystrophic muscles, approximately 70 Pax7^+^ satellite cells per condition were counted, and ZStacks images were taken (63X) for the Pax7^+^γHAX^+^ cells with the same exposure time. In cell cultures, images of at least 50-70 γHAX^+^ cells were taken (40X or 63X) at different Ztacks maintaining the same image acquisition conditions. Pax7^+^γHAX^+^ and Pax7^+^HP1γ^+^ were performed in two different tissue sections from at least three animals per condition. CD45^+^, F4/80^+^ and PDGFRα^+^ cells with positive or negative γHAX foci were counted in 15 independent fields (×63 magnification) from at least three different animals per strain and age and in two sections per animal. γHAX^+^ cells were related to the total number of cells (100%) and expressed as the percentage of positive cells.

SA-𝛽-gal-positive cells were detected by phase contrast microscopy. Quantification was performed by counting the positive cells in 10-15 independent fields (×20 magnification) and related to the total number of cells, in at least three independent experiments. MMP-10 and/or γHAX^+^ average intensity in quiescent satellite cells, FAPs and Lin-cells and myoblasts, from at least three independent experiments, were quantified in images from 20 independent fields (40x or ×63 magnification) taken at different Ztacks maintaining the same image acquisition conditions. When required, in each muscle-associated cell population, MMP-10^+^ cells were related to the total number of cells (100%) and expressed as the percentage of positive cells.

Detection and quantification of γH2AX foci was performed by using a plugin developed for Fiji/ImageJ, an open-source Java-based image processing software program (Schneider et al., 2012), modified by the Imaging Platform of CIMA (https://cima.unav.edu/en/investigacion/plataformas/imagen). First, a 3D region of interest (ROI) containing the cell to analyze was manually selected, using the information from all the channels. Then, the red channel containing the protein of interest was further processed to detect and quantify the foci. Background subtraction was performed applying a *rolling ball* algorithm, and a 3D median filter with a 2-voxel radius was applied for noise reduction. The resulting image is used to detect the foci by applying the 3D Object Counter plugin (thresholding and size filtering operations) developed for Fiji and based on the work by Bolte et al. (Bolte and Cordelières, 2006).

### Immunoblotting

Preparation of muscles and cell lysates and western blotting was carried out as described previously (Bobadilla et al., 2014). The following antibodies were used: anti-collagen IV (Abcam), anti-laminin (Sigma), anti-β-dystroglycan (Santa Cruz), anti-fibronectin (Santa Cruz), anti-MMP-10 (Acris; Abcam), anti-phosphorylated and total Rb (Cell Signalling), anti-γH2AX (Cell signalling), anti-β-tubulin (Promega) and anti-β-actin (Sigma).

### Quantitative PCR (qPCR)

Total RNA was isolated from muscles and purified using Trizol (Ambion). In FACS-sorted cells, total RNA was isolated and purified using the MagMAX *mir*Vana total RNA isolation Kit (Applied Biosystems) from up to 150.000 cells, following the manufacturer’s recommendations. RNA was reverse transcribed, and the cDNA amplified using PowerUp SYBR Green Master Mix (Applied Biosystems). Transcript levels were quantified by real-time PCR (QuantStudio™ 5 Real-Time PCR System, Applied Biosystems) with the following conditions: 94°C for 10 min followed by 40 cycles consisting of 60 s at 94°C, 60 s at 58°C, and 60 s at 72°C, followed by 10 min at 72°C. For each transcript, a specific calibration curve consisting of 1, 2, 5, 10 and 20 ng of cDNA isolated from heart, testis, stomach or small bowel of wild type mice was included to analyze expression of *mmp10*, *p16INK4a*, *cyclinA2*, *cyclinE1*, *cdc6*, *mcm3*, *lmnb1*, *mmp2*, *mmp9* and *timp1*. All measurements were related to this curve and then normalized to *gapdh*. Reactions were run in triplicate. Specific primers were designed (Primer3 program; version 0.4.0) for murine *mmp10* (forward, 5′-TGA​TCT​CCT​TTG​CAG​TTG​GA-3´; reverse, 5′-ATA​AAA​TCC​AGG​GCC​AGG​TG-3′), *p16INK4a* (forward, 5′-CAA​CGC​CCC​GAA​CTC​TTT-3´; reverse, 5′-CAG​TTC​GAA​TCT​GCA​CCG​TA-3′), *cyclinA2* (forward, 5′-AAG​AGA​ATG​TCA​ACC​CCG​AAA-3´; reverse, 5′- ACC​CGT​CGA​GTC​TTG​AGC​TT-3′), *cyclinE1* (forward, 5′- CAA​AGC​CCA​AGC​AAA​GAA​AG -3´; reverse, 5′-CCA​CTG​TCT​TTG​GAG​GCA​AT-3′), *mcm3* (forward, 5′-CCG​TGG​AGT​GGT​TTG​CAT​TG-3´; reverse, 5′- CCG​TGG​AGT​GGT​TTG​CAT​TG-3′), *mmp2* (forward, 5′-ATG​GCA​AGT​ATG​GCT​TCT​GC-3´; reverse, 5′-GTA​GGA​GGT​GCC​CTG​GAA​G-3′), *mmp9* (forward, 5′-AGA​CGA​CAT​AGA​CGG​CAT​CC-3; reverse, 5′-GTG​GTT​CAG​TTG​TGG​TGG​TG-3′), *timp1* (forward, 5′-CAC​AGA​CAG​CCT​TCT​GCA​AC-3´; reverse, 5′-TGGGGAACC CATGAATTTAG-3′), *gapdh* (forward, 5′- CAATGCATCCTGCACCAC -3´; reverse, 5′- CAG​TGA​TGG​CAT​GGA​CTG​TG-3′), *actb* (forward, 5′-GAC​GGC​CAG​GTC​ATC​ACT​AT-3´; reverse, 5′- CTT​CTG​CAT​CCT​GTC​AGC​AA-3′) and human *mmp10* (IDT, Hs.PT.58.38586852; Reference sequence: NM_002425) and *gapdh* (IDT, Hs.PT.39a.22214836; Reference sequence: NM_002046) genes. Comparisons between samples run in the same plate were performed by deltadeltaCT data. Data from samples run in different plates were showed as deltaCT, and statistical analyses comparing results were not carried out. Triplicates from at least three different samples per condition were analyzed.

### Microarray and single cell/nucleus RNA-sequencing analysis

Microarray data of quiescent satellite cells from young and aged mice were analyzed using GEO2R implemented in Gene Expression Omnibus (GEO) webpage (https://www.ncbi.nlm.nih.gov/geo/geo2r/). Raw microarray data are publicly available on GEO under the accession numbers GSE47177, GSE50821, GSE53728, GSE47401 and GSE81096 (Sinha et al., 1979; Liu et al., 2013; Price et al., 2014; Sousa-Victor et al., 2014; Lukjanenko et al., 2016). Gene expression heat maps were drawn using GENE-E software.

The 10X Genomics single cell RNA-sequencing (scRNA-seq) AnnData object from (Kimmel et al., 2021), including gene counts from skeletal muscle from two young (3 months) and two aged (20 months) mice was downloaded from https://myo.research.calicolabs.com/data. After conversion to a Seurat object, the expression of *Mmp10*, other metalloproteases, *Timps* and ECM-related genes was represented with the FeaturePlot and ViolinPlot functions for each individual mouse separately, considering the provided cell type annotation and UMAP coordinates. In addition, the 10X Genomics single nucleus RNA-seq SoupX corrected expression matrices from (Chemello et al., 2020) corresponding to TA muscles from a wild type and a dystrophic mouse (*Dmd* exon 51 deletion) were downloaded from NCBI (GSE156498). Downstream analyzes were performed with Seurat. Cells were filtered considering the same parameters as in their study, keeping cells expressing between 200 and 4,000 genes and less than 15,000 UMIs. The two samples were normalized, scaled and integrated using the SelectIntegrationFeatures, FindIntegrationAnchors and IntegrateData functions with default parameters. Cells were clustered considering the first 15 principal components and a resolution of 0.6 and reannotated using the markers from Figure 2; Supplementary Table S3 of their study. To get a similar cell type annotation, cluster 10 was subclustered with the FindSubCluster function considering a 0.15 resolution. Genes of interest were represented for each individual mouse separately using our re-annotation.

### Statistical analysis

All statistical analyses were performed using SPSS 15.0 (SPSS Inc.). The Shapiro-Wilk test was used to assess normal distribution. Variables were analyzed with the Mann–Whitney *U* test or t Student tests. All experiments were performed using at least three independent experiments per each condition. Data are expressed as mean ± SEM. *p* values <0.05 were considered to be statistically significant.

## Results

### Loss of MMP-10 induces aging-associated changes in the muscle of young mice

**FIGURE 1:**
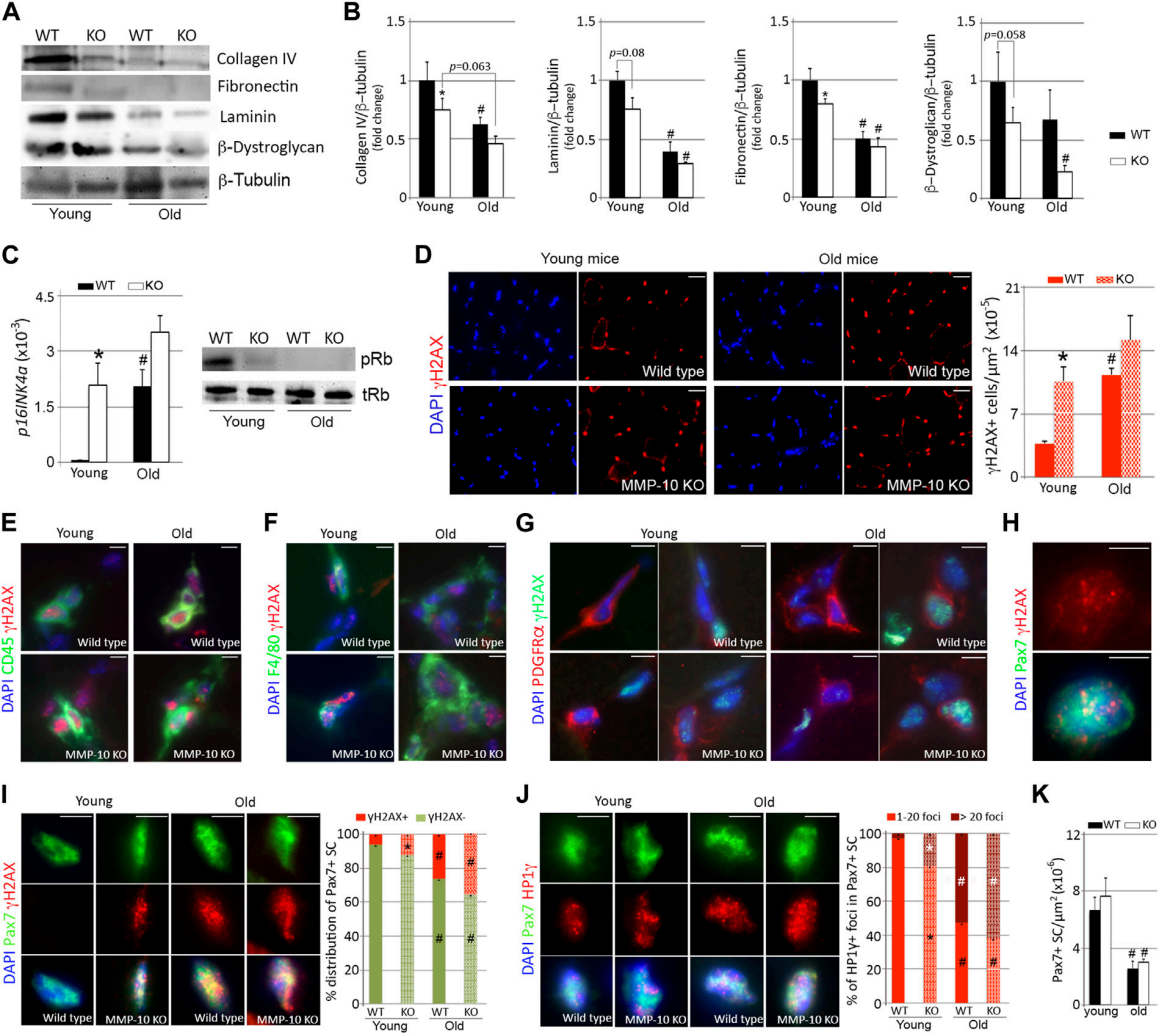
MMP-10 loss is associated to muscular aging and it confers aging features to the satellite cells. Representative western blots of the TA muscles from wild type and MMP-10 KO mice at 2 (young) or 18 (old) months of age **(A)**, using antibodies to proteins indicated, and quantification of ECM protein levels **(B)**. *p16INK4a* gene and pRb protein **(C)** expression in muscles of wild type and mutant mice at different ages. Representative muscle tissue sections of wild type and MMP-10 KO mice at 2 and 18 months of age immunostained for γH2AX **(D)**, CD45/γH2AX **(E)**, F4/80/γH2AX **(F)**, PDGRFα/γH2AX **(G)**, Pax7/γH2AX **(H, I)** and Pax7/HP1γ **(J)**. Graphs show total number of γH2AX^+^ cells in the muscles **(D)**, percentage of Pax7^+^ satellite cells containing positive γH2AX **(I)** and positive HP1γ foci **(J)**. Total number of Pax7^+^ satellite cells per μm^2^ in the TA muscles of wild type and KO mice at different ages **(K)**. DAPI was used to identify all nuclei. Scale bar: 40 μm. All numerical data were expressed as the mean ± SEM from at least three mice per genotype and age, including two independent tissue sections per mice. ^*^ designates significance between wild type and MMP-10 KO mice at the same age, while ^#^ defines significance between young and old mice of same origin, where ^*^ and ^#^ *p* < 0.05. mo, months; KO, MMP-10 knockout mice; WT, wild type mice; TA, *tibialis anterior*.

To investigate whether MMP-10 deficiency induces muscular aging through an ECM remodeling process, we examined the muscles of MMP-10 knockout (MMP-10 KO) mice, where MMP-10 is missing from all cells and tissues within these animals. We used MMP-10 KO and wild type mice at 2 (young) and 18 (old) months of age and focused on collagen type IV, fibronectin, laminin and *β*-dystroglycan ECM components, because their abundance in the niche and their close contact to satellite cells (Schüler et al., 2022) already suggested a link with satellite cell aging. MMP-10 loss and aging reduced the abundance of collagen type IV, fibronectin and laminin protein levels, while *β*-dystroglycan content underwent a decrease as consequence of MMP-10 deficiency but not as consequence of aging (Figures 1A, B). In addition, muscles of young MMP-10 KO mice showed upregulation of *p16* ^*Ink4a*^, de-phosphorylation of the retinoblastoma (Rb) and downregulation of downstream target genes, such as *CyclinA2*, *CyclinE1*, *Mcm3*, *Cdc6* and *Lmnb1* (Figure 1C; Supplementary Figure S1A). This was accompanied by an increase in cells containing the phosphorylated form of the histone γH2AX (Figure 1D), a marker of DNA damage that accumulates in aged cells (Kuilman et al., 2010). Leukocytes and macrophages were susceptible to age-related DNA damage but also to damage caused by MMP-10 deficiency (Figures 1E, F; Supplementary Figures S1B, 1C). However, neither aging and MMP-10 loss modified the number of FAPs with γH2AX foci, which was surprisingly high (Figure 1G; Supplementary Figure S1D).

Most of the changes that mimic the activation of p16/pRb senescence signaling in aged wild type mice was not more marked in aged MMP-10 KO mice (Figures 1A–G; Supplementary Figures S1A–1D). Because compensatory mechanisms triggered by a deficiency of MMP-10 can contribute to the aging-like ECM remodeling process, we analyzed gene expression of *Mmp2* and *Mmp9*, the most relevant MMPs in skeletal muscle, and *Timp1*, the main inhibitor of MMP-10. mRNA levels of *Mmp2* were upregulated in the muscles as consequence of aging but not as consequence of MMP-10 loss (Supplementary Figure S1E). In contrast, *Mmp9* and *Timp1* gene expression was higher in young MMP-10 KO mice than in wild type mice, reproducing to a faint extent the increase found in aged wild type mice (Supplementary Figure S1E).

Collectively, these findings support the link between MMP-10 and muscular aging and suggest that loss of MMP-10 in young mice changes the muscle ECM, conferring features of premature aging on the muscles.

### MMP-10 loss causes niche ECM changes with satellite cells acquiring features of aged cells

Satellite cells were also vulnerable to cellular damage by both MMP-10 deficiency and aging (Figures 1H, I). At a young age, the satellite cell compartment of MMP-10-deficient mice was composed of more Pax7^+^ satellite cells containing γH2AX^+^ foci, at twice the level of those found in wild type animals (Figure 1I). As expected, aging increased the presence of Pax7^+^γH2AX^+^ satellite cells both in wild type and MMP-10 KO mice (Figure 1I). Similarly, the senescence-associated heterochromatin histone HP1γ, an aging stressor unrelated to DNA damage (Gutiérrez‐Fernández et al., 2015), accumulated in the Pax7^+^ satellite cells of young deficient mice and spread further in those of aged mice (Figure 1J). Furthermore, the Pax7^+^ satellite cells from young MMP-10-deficient and old wild type mice present an increased nuclear volume size (Supplementary Figure S1F), a common feature of aged cells (Pathak et al., 2021). Interestingly, MMP-10 deficiency did not affect the stem cell pool as it did physiological aging, with comparable numbers of Pax7^+^ satellite cells being found in wild type and KO animals at the same age (Figure 1K). By co-immunostaining the muscle tissue sections for the myogenic commitment marker MyoD and Ki67, indicative of cell proliferation, we found that muscles of KO mice doubled the presence of MyoD^+^Ki67^-^ satellite cells, compared to wild type mice (Supplementary Figure S1G), suggesting stem cell phenotypic differences related to MMP-10 loss.

**FIGURE 2:**
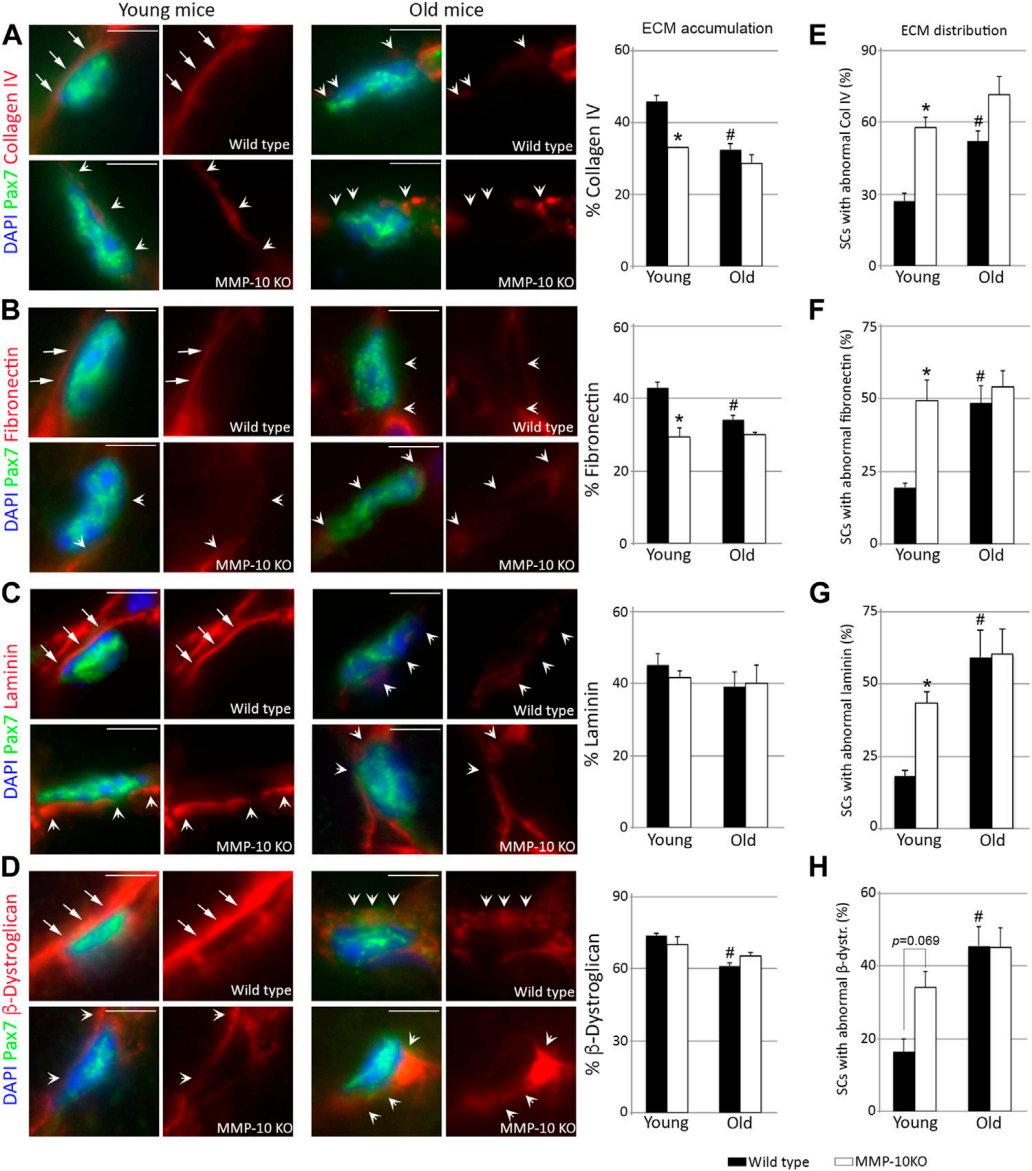
Loss of MMP-10 modifies the satellite cell niche ECM. Representative TA muscle sections of wild type and KO mice at 2 and 18 months of age co-immunostained for Pax7 and collagen IV **(A)**, fibronectin **(B)**, laminin **(C)** and *β*-dystroglican **(D)**. All MEC protein images were taken at the same exposure time. Arrows identified the continuity of the ECM proteins in the Pax7^+^ satellite cells from young wild type mice, while arrowheads highlight an abnormal ECM, with disruptions or rare accumulation of the proteins. DAPI was used to identify all nuclei. Scale bar: 5 μm. Graphs show the average accumulation of each protein in the niche ECM **(A–D)** and the percentage of Pax7^+^ satellite cells with abnormal protein distribution in the niche ECM **(E–H)**. All values were expressed as the mean ± SEM from three animals per condition, analyzing two independent tissue sections per mice. ^*^ designates significance between wild type and MMP-10 KO mice at the same age, while ^#^ defines significance between young and old mice of same origin (*p* < 0.05). KO, MMP-10 knockout mice; WT, wild type mice; SCs, satellite cells; *β*-dystr, *β*-dystroglican.

To obtain a comprehensive understanding of the consequences of MMP-10 loss at the satellite cell level, we examined the niche ECM. Aging and MMP-10 loss similarly reduced the abundance of collagen type IV and fibronectin surrounding the Pax7^+^ satellite cells (Figures 2A, B). However, the quantity of laminin in the niche ECM was unaltered in both wild type and MMP-10 KO mice at any age (Figure 2C), while *β*-dystroglican was reduced in old wild type mice (Figure 2D). Using a Z-stack analysis of immunostained muscle sections, we detected that most satellite cells from wild type mice at a young age rested on a continuous layer of collagen type IV and laminin, which completely covered their basal side (Figures 2A, C, E, G; Supplementary Figures S2A, 2B; arrows). In contrast, satellite cells of young mutants and aged animals exhibited a marked disorganization, showing a discontinuity and/or an unusual accumulation of these ECM components in their contact with the satellite cells (Figures 2A, C, E, G; Supplementary Figures S2A, 2B, arrowheads). Fibronectin was in continuous contact with the satellite cells in young wild type mice (Figures 2B, F; Supplementary Figure S3A; arrows). However, MMP-10 loss and aging markedly disrupted fibronectin organization, presenting a mesh-like arrangement in the stem cell niche with more and larger regions of abnormal accumulation and loss of continuity in the connections with the satellite cells (Figures 2B, F; Supplementary Figure S3A; arrowheads). Similarly, aging and MMP-10 loss produced small or large *β*-dystroglican disruptions and unusual accumulations in the satellite cell niche (Figures 2D, H; Supplementary Figure S3B; arrowheads), while its distribution was uniformly aligned with most young wild type satellite cells (Figures 2D, H; Supplementary Figure S3B; arrows).

These findings demonstrate that loss of MMP-10 induces aging-associated changes to the niche ECM, predisposing satellite cells to acquire features of aged cells, even before a decline in stem cell numbers is underway.

### Loss of MMP-10 leads young satellite cells to function as aged cells

**FIGURE 3:**
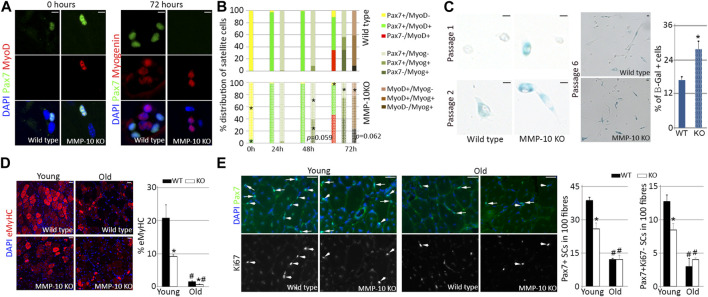
Satellite cells from young MMP-10 KO mice function as cells from aged mice. Representative images of single EDL myofibres isolated from young MMP-10 KO and wild type mice stained for Pax7 and MyoD or Myogenin, immediately after isolation or after 72 h in mitogen-rich media **(A)**. Distribution of the satellite cells according to the expression of different pairs of myogenic transcription factors, immediately after isolation and after 24, 48, and 72 h in mitogen-rich media **(B)**. Satellite cells from young KO and wild type mice stained for *β*-galactosidase after consecutive passages and percentage of senescent cells at passage 6 **(C)**. Representative images of the TA muscles of wild type and KO mice at different ages stained for eMyHC **(D)**, a protein expressed in fetal and newly regenerating fibers (Sacco et al., 2010), and Pax7/Ki67 **(E)** after two episodes of injury. Arrows and arrowheads indicate Pax7^+^Ki67^+^ and Pax7^+^Ki67^-^ satellite cells, respectively. DAPI was used to identify all nuclei. Scale bar: 5 μm **(A, C)**, 40 μm **(D, E)**. Percentage of regenerating fibers **(D)** and number of Pax7^+^ and Pax7^+^Ki67^-^ satellite cells in 100 fibers **(E)** in young and old wild type and KO mice 7 days after last episode of damage. Values are presented as the average of at least three independent experiments (mean ± SEM). ^*^ and ^#^ designate significance (*p* < 0.05) between wild type and KO mice at the same age or between adult and aged mice of same strain, respectively. KO, MMP-10 knockout mice; WT, wild type mice; *β*-Gal, *β*-galactosidase; eMyHC, embryonic Myosin; SCs, satellite cells; TA, *tibialis anterior*; EDL, *extensor digitorum longus*.

If MMP-10 loss produces signs of aging in the satellite cells of young mice, the transition of the cells between quiescence, commitment, differentiation and self-renewal would be altered. Therefore, we used the single myofiber system (Bobadilla et al., 2014) to explore whether MMP-10 deficiency affects satellite cell fates. Immediately after isolation, few Pax7^+^ satellite cells from MMP-10 KO fibers were positive for the myogenic commitment marker MyoD, indicative of cells escaping from quiescence, compared to the satellite cells of wild type origin (Figures 3A, B). By 24–48 h in mitogen-rich media, fiber-associated satellite cells from KO mice began undergoing myogenic differentiation by expressing myogenin, with some cells losing Pax7, in comparison to those cells in wild type fibers (Figure 3B). By 72 h, the majority of satellite cells from MMP-10-deficient mice expressed myogenin and adopted an advanced differentiation pattern, with a decline in the number of Pax7^+^MyoD^−^ self-renewed satellite cells, as compared to control mice (Figures 3A, B). The behavior of MMP-10-deficient satellite cells resembled to some extent that of aged satellite cells (Chakkalakal et al., 2012; Bernet et al., 2014), which is further characterized by susceptibility to senescence (Sousa-Victor et al., 2014). In an attempt to confirm this last statement, satellite cells from young mice were removed from their native environment, plated into Matrigel and forced to age by consecutive passages under proliferative conditions. This situation led satellite cells from MMP-10 deficient mice to enter senescence earlier than the cells of wild type origin (Figure 3C) as determined by the accumulation of *β*-galactosidase (β-Gal), a conclusive feature of cellular senescence (Dimri et al., 1995). These outcomes show that MMP-10 deficiency breaks satellite cell quiescence, favoring myogenic differentiation over self-renewal, with more satellite cells-derived myoblasts predisposed to senescence under replicative pressure.

If the satellite cells of young MMP-10 KO mice have signs of premature aging, then they should undergo a striking decline in their response to damage under regenerative pressure (Sousa-Victor et al., 2014). To address this hypothesis, muscles of young and old mice from mutant and wild type origin were subjected to two cycles of damage by intramuscular injection of notexin. Seven days after the last episode of injury, staining for the embryonic isoform of MyHC, a marker of the earliest stage of muscle fiber formation, revealed that young MMP-10 KO muscles had fewer immature fibers than wild type mice at the same age (Figure 3D). Aging weakened regeneration efficiency and this situation was exacerbated in MMP-10 KO mice (Figure 3D). Importantly, MMP-10 loss and aging had the same effect on the numbers of total Pax7^+^ and Pax7^+^Ki67^-^ self-renewed satellite cells, which were significantly decreased after regenerative pressure (Figure 3E). Thus, our data support that the satellite cells from young mice deficient for MMP-10 are unable to properly participate in the muscle repair response due to a functional decline.

MMP-10 loss causes intrinsic and extrinsic changes associated with a decline in satellite cell function.

**FIGURE 4:**
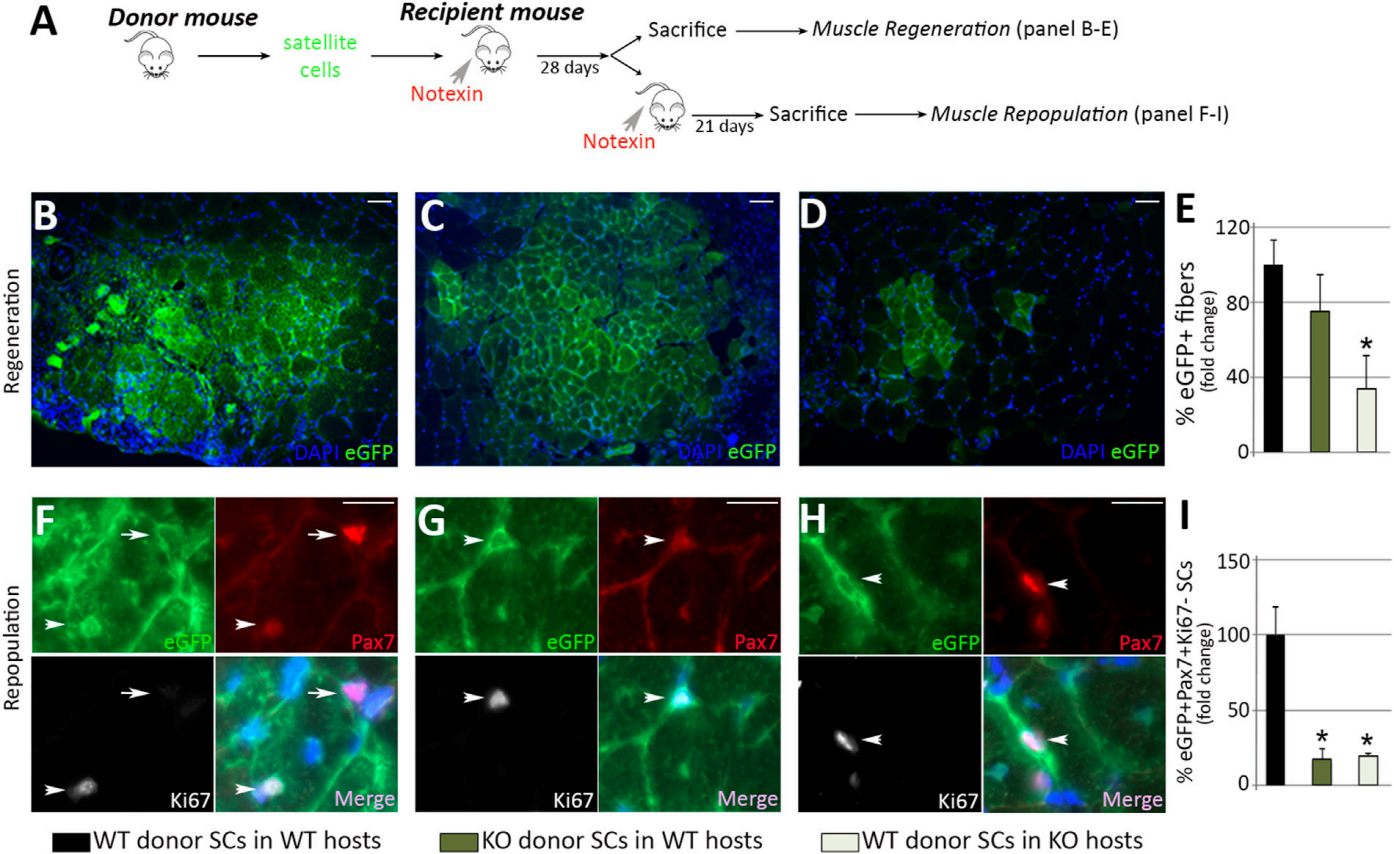
Loss of MMP-10 induces intrinsic and extrinsic changes in the satellite cells at a young age. Experimental schematic outlining the transplantation of satellite cells isolated from donor mice into the TA muscles of pre-injured recipient mice **(A)**. Representative images of recipient muscles stained for GFP **(B–D)** and percentage of new GFP^+^ regenerating fibers of donor origin at 28 days after cell delivery **(E)**. TA sections of recipient mice stained for GFP, Pax7 and Ki67 **(F–H)** and total number of GFP^+^Pax7^+^Ki67^-^ quiescent satellite cells of donor origin **(I)** 3 weeks after inducing damage in recipients. Arrows and arrowheads identify Pax7^+^Ki67^-^ and Pax7^+^Ki67^+^ satellite cells of donor origin (GFP^+^), respectively. DAPI was used to identify all nuclei. Scale bar: 50 μm **(B, C, D)**, 20 μm **(F, G, H)**. B, F and black bars represent wild type donor satellite cells delivered into wild type recipients (control group); C, G and dark green bars designate MMP-10 KO donor cells transferred into wild type mice; D, H and pale green bars represent satellite cells of wild type origin transplanted into KO recipients. All measurements were related to those from the control group, which were assigned as 100%. Data are expressed as the mean ± SEM from at least four engraftments per group. ^*^ designates significance between control and experimental groups where ^*^ *p* < 0.05. WT, wild type mice; KO, MMP-10 knock-out mice; SCs, satellite cells; TA, *tibialis anterior*.

Our findings suggest that MMP-10 loss induces both intrinsic and extrinsic changes to satellite cells. To clarify whether these effects are related to deficiency of MMP-10, we carried out cell transfer assays by challenging the ability of the cells to proliferate, differentiate and self-renew in recipient mice (Figure 4A). To investigate whether MMP-10 loss induces intrinsic defects in muscle stem cells, 10.000 satellite cells from wild type and MMP-10 KO mice at a young age, labelled with green fluorescent protein (GFP), were delivered into pre-injured muscles of young wild type recipients. In parallel, to investigate whether deficiency of MMP-10 involves satellite cell-extrinsic changes as a driving force for intrinsic defects, the same numbers of GFP^+^ satellite cells from young wild type mice were transplanted into notexin-treated muscles of young MMP-10 KO mice. After 28 days, both wild type and MMP-10 KO populations of donor satellite cells efficiently regenerated the wild type recipients (Figures 4B, C, E). However, the number of new GFP^+^ fibers of wild type origin was significantly reduced in MMP-10 KO recipient mice, showing less donor contribution to host muscle fiber differentiation (Figures 4D, E).

At 28 days after transplantation, some recipients were subjected to a new episode of injury to analyze the self-renewal ability of donor cells. Three weeks after damage, the capacity of Pax7^+^GFP^+^ engrafted satellite cells of mutant origin to reoccupy the stem cell niche of wild type recipients was substantially reduced, compared to engrafted satellite cells of wild type origin (Figures 4F, G, I). Similarly, engrafted satellite cells from wild type mice were less capable of reoccupying the niche of MMP-10 KO recipients (Figures 4H, I).

Taken together, these results indicate that loss of MMP-10 induces intrinsic defects in muscle stem cells, which act as committed progenitors limited to differentiation rather than true stem cells with a long-term potential for self-renewal, resembling the behavior that most satellite cells exhibit in aged animals (Chakkalakal et al., 2012). Furthermore, these findings demonstrate that the ability of young satellite cells to repair muscle depends on the environment of recipients, and support MMP-10 loss as an extrinsic component responsible for declines in satellite cell function.

Quiescent satellite cells do not express MMP-10 at the transcriptional level but the protein accumulates in muscles.

**FIGURE 5:**
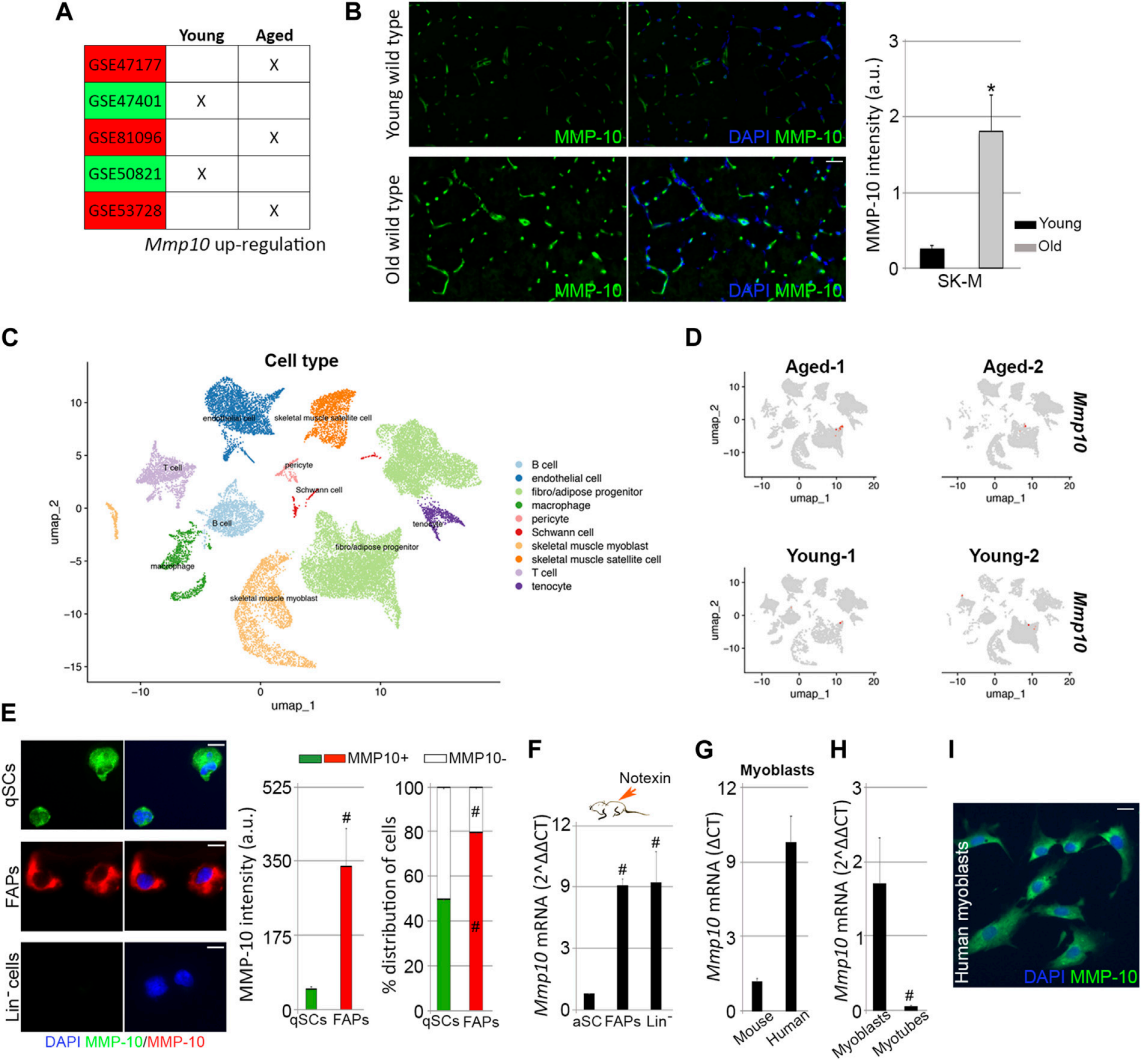
MMP-10 mRNA and protein levels. Upregulated *Mmp10* transcript levels associated with published microarrays performed in quiescent satellite cells sorted from young and old mice **(A)**. Representative images of muscle tissue sections (scale bar: 40 μm) from wild type mice at 2 and 18 months of age, immunostained for MMP-10 and quantification of MMP-10 average intensity **(B)**. UMAP representations of cell type populations within the skeletal muscles from young and aged mice (retrieved from Kimmel et al., 2021) **(C)**. UMAPs represent the normalized gene expression of *Mmp10* for each sample separately **(D)**. Representative images of FACS-sorted quiescent satellite cells, FAPs and Lin^−^ cells isolated from young wild type mice immunostained for MMP-10 (scale bar: 20 μm), quantification of average intensity of MMP-10 and percentage of cells expressing MMP-10 **(E)**. Expression of *Mmp10* mRNA levels in FACS-sorted activated satellite cells, FAPs and Lin^−^ cells isolated from muscles of young wild type mice 3 days after notexin injection **(F)**. Quantification of *Mmp10* gene levels in myoblasts of human origin **(G)** and in myoblasts and myotubes from wild type mice **(G, H)**. Representative image of human myoblasts stained for MMP-10 **(I)**. Bars represent the mean ± SEM of at least three independent samples per condition. ^*^ and ^#^ designate significance (*p* < 0.05) between wild type and *mdx* cells from same subtype or between cell subpopulations from same strain, respectively. SK-M, skeletal muscle; a.u, arbitrary units; UMAP, uniform manifold approximation and projection; qSCs, quiescent satellite cells; aSCs, activated satellite cells; Lin^−^, lineage negative cells; FAPs, fibro-adipogenic progenitor cells.

Our data suggest a relationship between MMP-10 and aging. To address this connection, *Mmp10* gene expression was analyzed by qPCR in FACS-sorted quiescent satellite cells from young and aged wild type mice. *Mmp10* gene was not found in satellite cells from old mice. However, *Mmp10* transcripts were neither detected in the cells obtained from mice at a young age. For the purpose of further investigating the link between MMP-10 deficiency and satellite cell aging, we examined publicly available satellite cell microarray data sets from young and aged mice using gene set enrichment analysis (GSE47177 (Liu et al., 2013), GSE50821 (Sinha et al., 1979), GSE53728 (Sousa-Victor et al., 2014), GSE47401 (Price et al., 2014) and GSE81096 (Lukjanenko et al., 2016). However, no conclusive data was found, with enrichment scores of data sets revealing that quiescent satellite cells expressed very low levels of *Mmp10* gene (Figure 5A; Supplementary Figure S4). In two data sets, *Mmp10* expression was higher in the satellite cells from young mice, while in three data sets the contrary result was observed (Supplementary Figure S4). Given the existence of compensatory mechanisms between MMP members, we analyzed the expression of *Mmp2*, *Mmp3* and *Mmp9* genes, as well as that of their inhibitors (*Timp1*, *Timp2*, *Timp3* and *Timp4*), finding low expression levels and discrepancies between data sets (Supplementary Figure S4). Similarly, all five data sets showed differences in the low expression of *Lama*, *Col4*, *Fn* and *Dag* genes (Supplementary Figure S4), which encode the proteins we have found affected by MMP-10 deficiency and aging. The disparity between data sets may be associated with differences with respect to the experimental conditions. However, it can also suggest that the remodeling of the niche ECM under physiological aging is a dynamic process that requires a small continuous change rather than a big sudden one in ECM-associated genes, as it has been shown before (Lukjanenko et al., 2016).

In order to understand the gene expression results, the protein levels of MMP-10 were analyzed in the muscle tissue sections from young and old wild type mice. By immunostaining we confirmed our previous results (Bobadilla et al., 2014), finding that young muscles expressed MMP-10 protein (Figure 5B). However, protein levels were upregulated by aging (Figure 5B). We next investigated the contribution of muscle-associated cell subtypes to the presence of MMP-10 protein in the muscles, which would further explain the phenotype developed by the MMP-10 KO mice. *Mmp10* gene expression was analyzed by qPCR in FACS-sorted FAPs and Lin^−^ cells, which included hematopoietic, immune and endothelial cells, from young and old mice. Again, no *Mmp10* expression was detected. Additionally, taking advantage of Kimmel´s published single cell-RNA sequencing data sets (Kimmel et al., 2021), we reproduced the cell population subtypes within the skeletal muscles of wild type mice at a young and old age (Figure 5C), finding no *Mmp10* transcripts at any cell subgroup (Figure 5D), independently of the age of the animals. Interestingly, ECM-associated genes (*Mmp2*, *Mmp3*, *Mmp9*, *Timp1*, *Timp2*, *Timp3*, *Timp4*, *Lama*, *Col4*, *Fn* and *Dag*) were expressed at very low levels by the different cell subpopulations, encountering no differences between samples from young and old mice (Supplementary Figure S5). These results were surprising because the muscle ECM is composed of these structural proteins and ECM regulators (Ogura et al., 2014; Loreti and Sacco, 2022b), which indeed are deregulated in aged muscles.

### Satellite cells express MMP-10 at the transcriptional level under replicative pressure

Since our data showed that muscle-resident cells, included the quiescent satellite cells, do not express *Mmp10* gene but the protein is present in the muscles, we questioned the origin of MMP-10. Post-transcriptional control and post-translational modifications can cause the absence of a direct correlation between mRNA and protein levels. Thus, expression of MMP-10 was analyzed at the protein level in FACS-sorted quiescent satellite cells, FAPs and Lin^−^ cells from young wild type mice, finding that the protease was present in quiescent satellite cells and FAPs but not in Lin^−^ cells (Figure 5E). Interestingly, FAPs contribution to MMP-10 protein was higher than quiescent satellite cells (Figure 5E). Among the quiescent satellite cells, MMP-10 protein was detected in 50% of the cells, while 80% of the FAPs were positive for MMP-10 (Figure 5E). We next analyzed whether the presence of MMP-10 modifies the susceptibility of the cells to accumulate damaged DNA. Only 10% of the total quiescent satellite cells were positive for γH2AX (Supplementary Figures S6A, 6B), in agreement with our previous data (Figure 1I). These γH2AX^+^ cells were mostly composed of MMP-10 negative satellite cells (Supplementary Figure S6C), which had the tendency to accumulate more damaged DNA than those satellite cells expressing the protease (Supplementary Figure S6D). Similarly, MMP-10 negative FAPs were more predisposed to accumulate damaged DNA than MMP-10 positive FAPs (Supplementary Figures S6E–6G). These results, further than confirming the heterogeneity among these cell subtypes, which may explain the low (or undetectable) *Mmp10* gene expression levels found in our analyses, support the connection between MMP-10 loss and aging.

We kept on inquiring about the source of MMP-10 in the skeletal muscles and in the satellite cells, suspecting that activated/proliferating cells within the muscle can supply the protein by activating the transcriptional machinery. To investigate this idea, the hind limb muscles of adult wild type mice were subjected to notexin injections. Three days later, animals were sacrificed and Itga7^+^/CD34^-^ activated satellite cells, FAPs and Lin^−^ cells isolated from the muscles by FACS. Under these experimental conditions, *Mmp10* gene expression was detected in activated satellite cells, and it was greatly upregulated in FAPs and Lin^−^ cells (Figure 5F). According to these findings, *Mmp10* transcripts were detected in satellite cell-derived myoblasts from young wild type mice (Figure 5G), with gene expression levels decreasing as myogenic progenitors differentiated into myotubes (Figure 5H). Consistent with this, myoblasts from healthy people expressed the protease at both the transcriptional and protein level (Figures 5G, I).

These findings showed that satellite cells express *Mmp10* by activating the transcriptional machinery under damage demand, with other muscle-associated cells contributing to muscle, and satellite cell, supply requirements.

### MMP-10 modulation regulates satellite cell aging

**FIGURE 6:**
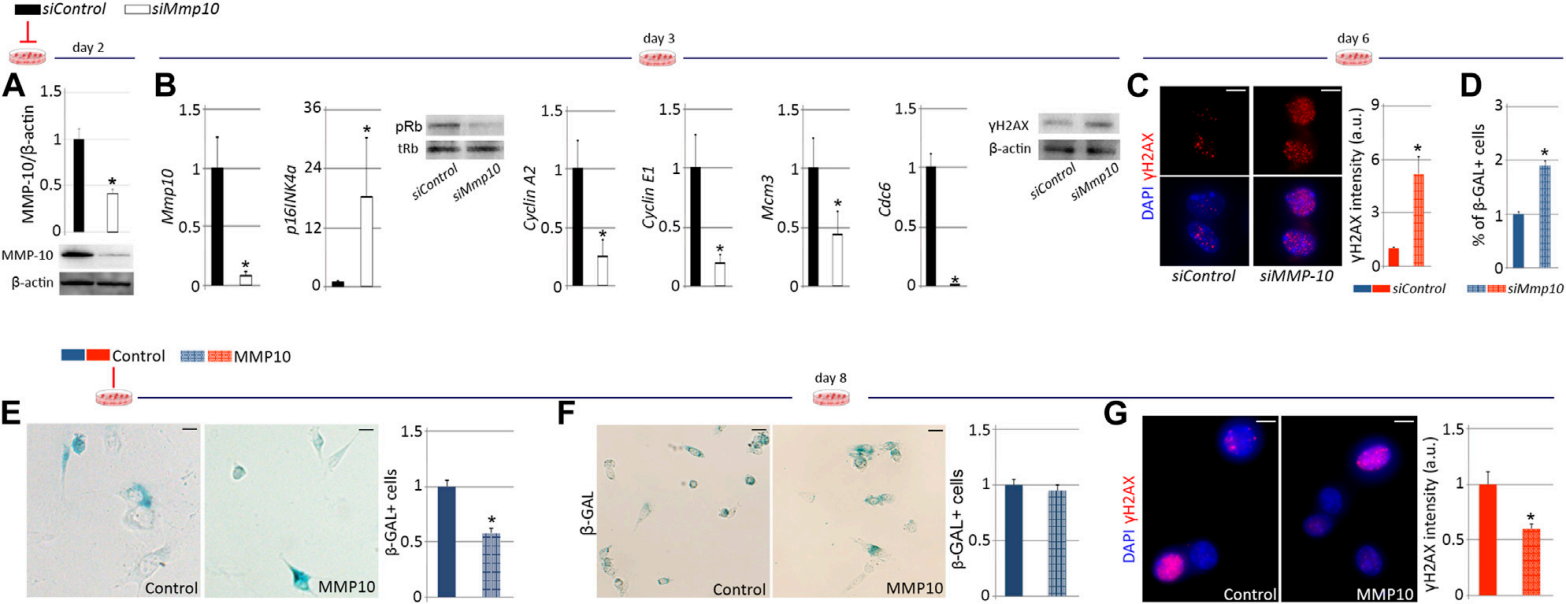
MMP-10 downregulation predisposes satellite cells to premature senescence. Protein levels of *Mmp10* in *siMMP10* and *siControl*-treated satellite cells isolated from young wild type mice, normalized to *β*-actin, two **(A)** and 3 days **(B)** after RNA silencing. *Mmp10*, *p16*, *CyclinA2*, *CyclinE1*, *Mcm3* and *Cdc6* mRNA expression levels in *siMMP10*-treated satellite cells related to *siControl*-treated cells and representative blots of *siRNA*-treated satellite cells stained for Rb and γH2AX proteins **(B)**, 3 days after MMP-10 downregulation. Representative images of *siMmp10* and *siControl*-transfected satellite cells stained for γH2AX and quantification of damaged DNA accumulation **(C)**, 6 days after MMP-10 silencing. Quantification of *siMMP10*-treated and *siControl*-satellite cells entering senescence **(D)**. Representative images and quantification of MMP10-treated and untreated-satellite cells isolated from young **(E)** and old **(F)** wild type mice, entering senescence. Satellite cells of old wild type origin immunostained for γH2AX and average intensity of γH2AX marks 8 days after addition of MMP-10 to the culture media **(G)**. Scale bar: 5 μm. Numerical data in *siMMP-10* and MMP-10-treated satellite cells were expressed as a fold change compared to *siControl* and non-treated cells, respectively. Data were expressed as the mean ± SEM of three animals per condition. ^*^ designates significance between control and experimental groups where ^*^ *p* < 0.05. pRb, phosphorylated retinoblastoma; tRb, total retinoblastoma; *β*-Gal, *β*-Galactosidase; a.u, arbitrary units.

To specifically delineate the effects of MMP-10 loss on satellite cell aging, we used an *in vitro* cell culture system. FACS-sorted quiescent satellite cells from young wild type mice were plated in Matrigel and cultured in mitogen-rich media, inducing satellite cell activation and proliferation. One day later, satellite cells were treated with a *siRNA* against *Mmp10* mRNA (*siMMP-10*) for two consecutive days to inhibit its expression at the mRNA and thus at the protein level (Figure 6A). Under proliferative cell culture conditions (∼50% cellular confluence), *Mmp-10* downregulated satellite cells activated the p16^INK4a^/pRb signaling pathway and accumulated damaged DNA (Figure 6B), with more cells entering deep senescence (Figures 6C, D), as compared to *siControl*-treated satellite cells. Importantly, MMP-10 downregulation did not result in activation of compensatory mechanisms (Supplementary Figure S7). These findings suggest that, independently of extrinsic alterations related to overall MMP-10 ablation, downregulation of this protease induces changes in satellite cells associated with premature aging.

If decreases in MMP-10 precipitate satellite cell aging, it could be hypothesized that addition of MMP-10 would delay and/or revert this process. To investigate this idea, satellite cells from young wild type mice were treated with MMP-10 and cultured under replicative pressure for 8 days *β*-Gal staining revealed that addition of MMP-10 to the growth media significantly reduced the number of satellite cells entering permanent cell cycle arrest, in comparison with untreated control cells (Figure 6E). We extended these experiments to satellite cells isolated from old wild type mice, finding that exogenous MMP-10 could not prevent aged satellite cells from entering senescence (Figure 6F) but did significantly diminish cellular damage (Figure 6G), compared to un-treated aged cells. These data suggest that MMP-10 can consistently slow the onset of senescence of satellite cells whenever they are not committed to this process. Interestingly, MMP-10 protects aged satellite cells from damage accumulation.

### MMP-10 treatment rescues dystrophic satellite cells from cellular damage

**FIGURE 7:**
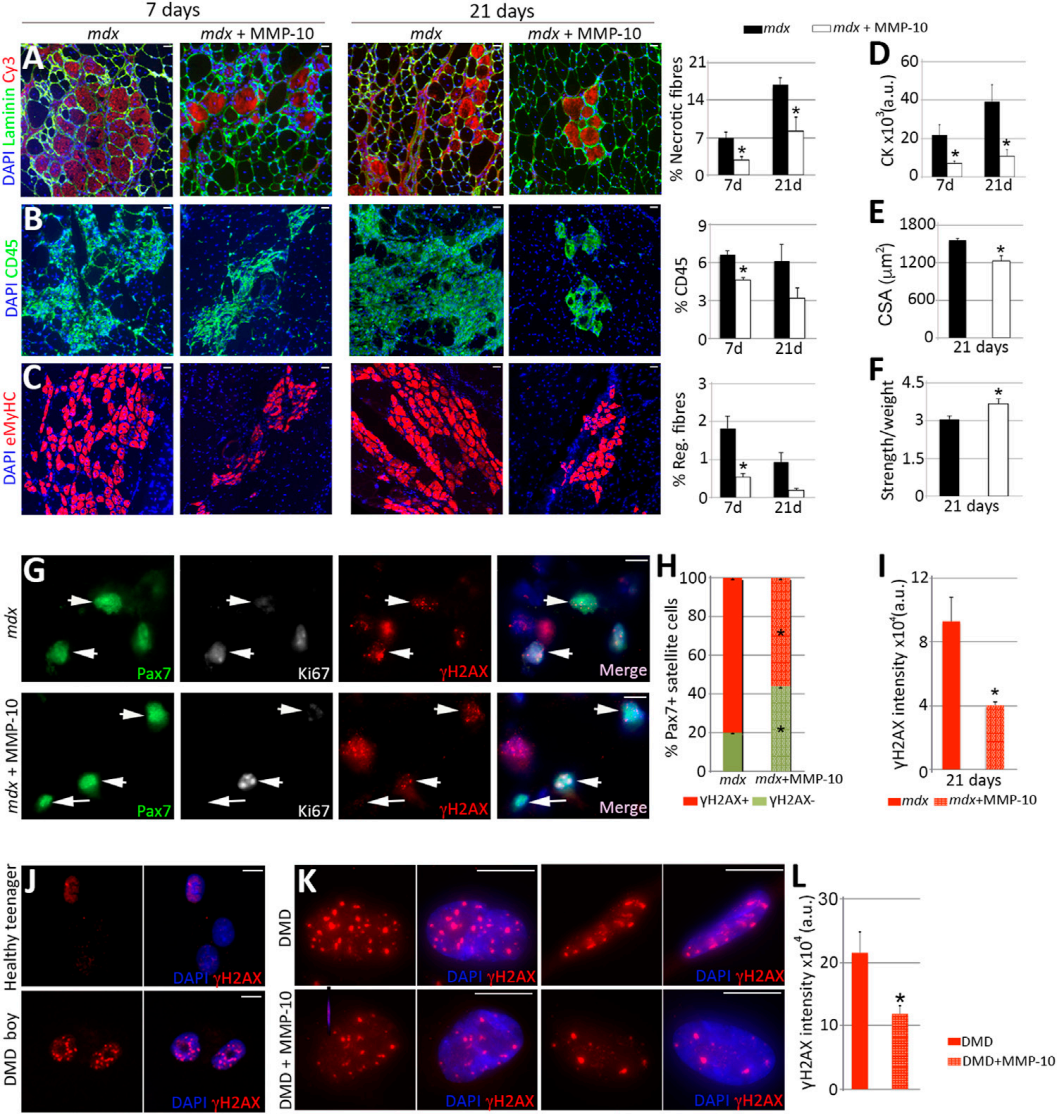
MMP-10 improves dystrophic phenotypes. **(A–I)** Representative images of *gastrocnemius* muscles from control and MMP-10-treated *mdx* mice immunostained by using antibodies to the proteins indicated 7 and 21 days after treatment administration. Necrotic fibers **(A)**, immune cells **(B)** and new regenerating fibers **(C)** localized in muscles of control and treated *mdx* mice after MMP-10 delivery. CK serum levels **(D)**, CSA **(E)** and muscle strength **(F)** in the experimental groups. TA muscles of control and MMP-10-treated *mdx* mice co-immunostained for Pax7, Ki67 and γH2AX **(G)**. Arrowheads identify Pax7^+^γH2AX^+^ satellite cells, while arrows point Pax7^+^Ki67^−^γH2AX^−^ satellite cells. Percentage of Pax7^+^γH2AX^+^ satellite cells **(H)** and average γH2AX accumulation in Pax7^+^γH2AX^+^ satellite cells **(I)** analysed in muscles from control and MMP-10-treated *mdx* mice after 21 days. Bars represent the mean ± SEM of at least three animals per condition. (**J–L)** Representative images of myogenic cells derived from a 19 years-old healthy boy and a 5 years-old Duchenne patient stained for γH2AX **(J)**. Human dystrophic muscle cells stained for γH2AX after 8 days in culture with or without MMP-10 **(K)**. γH2AX intensity accumulation **(L)** in treated and untreated human cells. Scale bar: 40 μm **(A–C, G)**, 5 μm **(J, K)**. Bars in L show the mean ± SEM of four independent experiments carried out from same human cells. ^*^ designates significance between experimental groups, where ^*^ *p* < 0.05. CK, Creatine Kinase; CSA, Cross Sectional Area; a.u, arbitrary units; DMD, Duchenne muscular dystrophy.

High levels of stressors, such as damaged DNA, may originate from consecutive injuries during life or from a decline in repair capacity (Allamand and Campbell, 2000; Oh et al., 2014). Thus, we considered that MMP-10 might be beneficial in a context of impaired muscle regeneration such as muscular dystrophy, where satellite cells are unable to appropriately maintain the muscle repair response. To address this idea, we employed the Duchenne muscular dystrophy mouse model (*mdx*), which lacks dystrophin expression due to a nonsense mutation (Allamand and Campbell, 2000). As expected, dystrophic muscles had more Pax7^+^ satellite cells with damaged DNA than muscles from wild type animals at the same age (Supplementary Figure S8A), according to the accumulation of γH2AX in stem cells linked to replicative stress (Rossi et al., 2007; Flach et al., 2014) and according to the frequently accumulation of *β*-Gal in the cytoplasm of dystrophic satellite cells (Zhang et al., 1979). To investigate the effect of MMP-10 in muscular disease progression, a single dose of MMP-10 was systemically injected into the *mdx* mice, since delivered MMP-10 can cross the endothelial barrier (Roncal et al., 2017) and could reach muscles to exert its effect. Seven days later, the hind limb muscles of the MMP-10-treated *mdx* mice had fewer necrotic fibers, lower cell infiltration and a reduced number of regenerating myofibers than untreated-*mdx* animals (Figures 7A–C; Supplementary Figures S8B–8G). We extended the single MMP-10 treatment to 21 days, finding similar results (Figures 7A–C; Supplementary Figures S8B–8G) including a reduction of creatine kinase serum levels (Figure 7D). In addition, the long-term MMP-10 regimen resulted in functional rescue, reducing the cross-sectional area of the myofibers (Figure 7E), which is consistent with recovery from the hypertrophic phenotype of dystrophic mice, and improving muscle strength (Figure 7F).

To explain the benefit of MMP-10 in the *mdx* mice, we examined the phenotype of satellite cells. Three weeks after MMP-10 delivery, the muscles of *mdx* mice had decreased numbers of Pax7^+^γH2AX^−^ satellite cells (Figures 7G, H, arrows) and reduced damaged DNA accumulation in those cells with γH2AX marks, compared to untreated dystrophic mice (Figures 7G, I, arrowheads). Notably, addition of MMP-10 increased the presence of Pax7^+^Ki67^-^ satellite cells (Supplementary Figure S8H) without damaged DNA (Figure 7G, arrows; Supplementary Figure S8I), suggesting that the presence of MMP-10 modulates the ECM and this situation will enable cells to spend more time in a quiescent stage, avoiding replicative stress. Thus, our findings indicate that MMP-10 treatment reduces cellular damage in dystrophic satellite cells, protecting satellite cell stemness and preventing muscle from continue damage. We further assessed the potential of MMP-10 for clinical application, considering that the *mdx* mouse model presents some limitations as compared to DMD patients (Yucel et al., 2018). As we suspected, human myogenic cells obtained from a DMD boy had more γH2AX marks than myogenic cells from a healthy teenager (Figure 7J). To evaluate the effect of MMP-10 in human dystrophic myogenic cells, MMP-10 was added to the growth media for 8 days. MMP-10-treated cells significantly decreased damaged DNA accumulation by diminishing the abundance of positive γH2AX foci (Figures 7K, L), as compared to un-treated cells. This outcome supports the notion of MMP-10 as a viable therapeutic strategy to target dystrophic satellite cells to reduce cell stress and suppress excess muscle regeneration.

### Dystrophic condition reduces MMP-10 expression in the satellite cells under proliferative pressure

**FIGURE 8:**
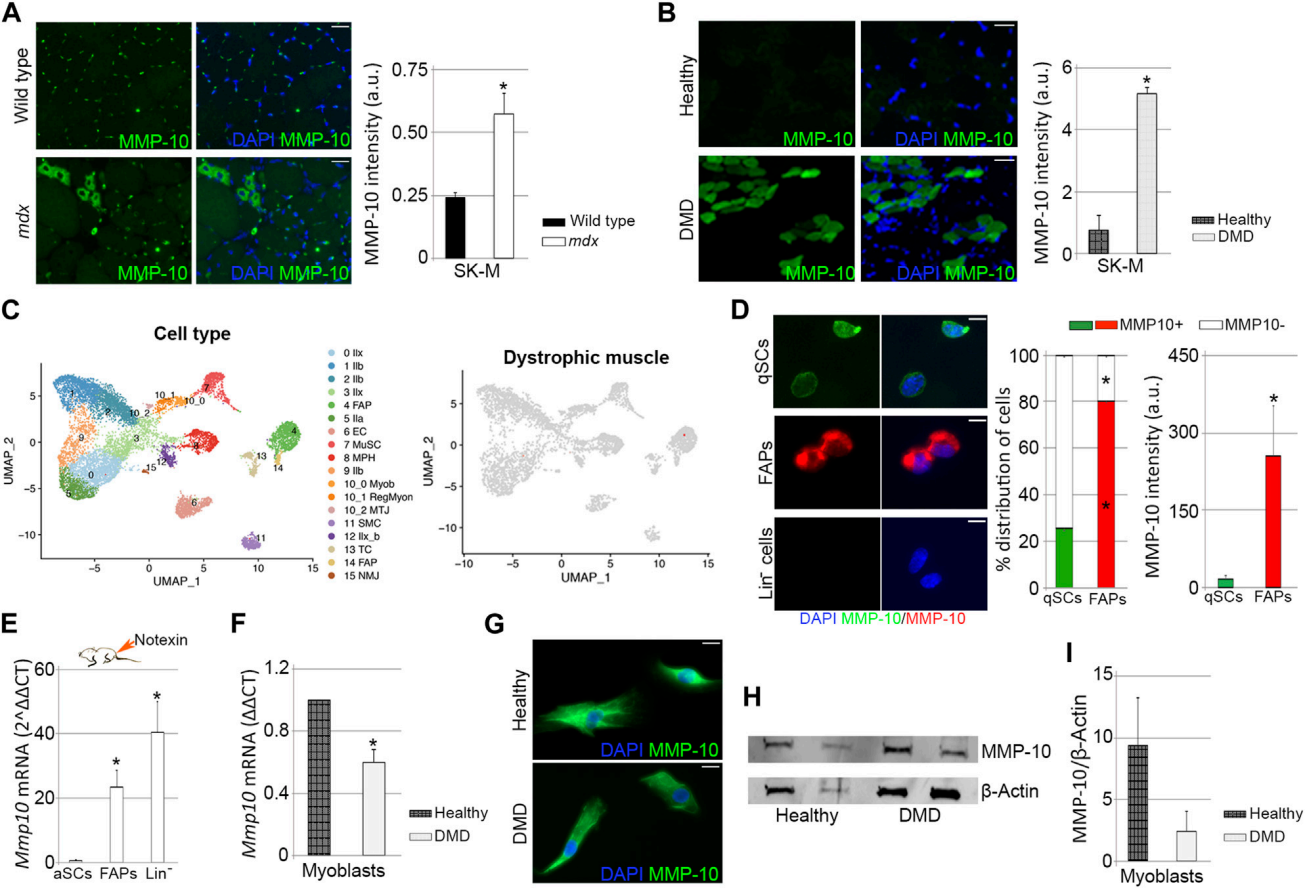
Expression of MMP-10 in dystrophic and healthy muscles. Representative images of muscle tissue sections (scale bar: 40 μm), from wild type and *mdx* mice **(A)** and from healthy people and DMD patients **(B)**, immunostained for MMP-10 and quantification of MMP-10 average intensity. UMAP representations of cell type populations within the skeletal muscles of dystrophic mice retrieved from Chemello et al., 2020 **(C)**. On the right, UMAPs represent the normalized gene expression of *Mmp10*. Representative images of FACS-sorted quiescent satellite cells, FAPs and Lin^−^ cells isolated from *mdx* mice immunostained for MMP-10 (scale bar: 20 μm), quantification of percentage of cells expressing MMP-10 and average intensity of MMP-10 **(D)**. Expression of *Mmp10* mRNA levels in FACS-sorted activated satellite cells, FAPs and Lin^−^ cells isolated from muscles of *mdx* mice 3 days after notexin injection **(E)**. mRNA expression levels of MMP-10 in myoblasts isolated from human biopsies of healthy people and DMD patients **(F)**. Representative images of human myoblasts (scale bar: 40 μm) immunostained for MMP-10 **(G)** and quantification of MMP-10 protein accumulation based on western-blot data **(H, I)**. Bars represent the mean ± SEM of at least three different samples per condition, where ^*^ designates significance (*p* < 0.05) between the experimental groups. a.u, arbitrary units; SK-M, skeletal muscle; DMD, Duchenne muscular dystrophy; IIb, type IIb myonuclei; IIx, type IIx myonuclei; IIa, type IIa myonuclei; IIx_b, type IIx_b myonuclei EC, endothelial cell; MuSC, muscle satellite cells; MPH, macrophages; Myob, myoblasts; RegMyon, regenerative myonuclei; MTJ, myotendinous junction myonuclei; SMS, smooth muscle cells; TC, tenocytes; NMJ, neuromuscular junction myonuclei; FAP, fibro-adipogenic progenitor cells; UMAP, uniform manifold approximation and projection; Lin^−^, linage negative cells; qSC, quiescent satellite cells; aSC, activated satellite cells.

In order to explain the benefits of MMP-10 administration into the *mdx* mice, we analyzed the presence of MMP-10 by immunostaining, verifying that dystrophic condition induced protein accumulation (Bobadilla et al., 2014), both in mononuclear cells and in eMyHC^+^ fibers, compared to wild type mice (Figure 8A; Supplementary Figures S9A, 9B). Similarly, muscles from DMD patients had higher MMP-10 content than muscles from healthy donors (Figure 8B), with the protein predominantly accumulated in new regenerating fibers (Supplementary Figures S9C–9E; arrows).

To understand the increase of MMP-10 in the dystrophic muscles, we assessed *Mmp10* gene expression by qPCR in FACS-sorted quiescent satellite cells, FAPS and Lin^−^ cells from the *mdx* mice, finding no transcripts in any cell population. In order to confirm this data, we next reproduced the cell population subtypes within dystrophic muscles by using published single nucleus RNA-sequencing data sets (Chemello et al., 2020). As well as in wild type muscles, there were no cells expressing *Mmp10* (Figure 8C; Supplementary Figure S9F). Additionally, genes related to the ECM (*Mmp2*, *Mmp3*, *Mmp9*, *Timp1*, *Timp2*, *Timp3*, *Timp4*, *Lama*, *Col4*, *Fn* and *Dag*) were detected at low levels in dystrophic muscle-associated cell subpopulations, finding no differences with levels expressed by wild type cell subtypes (Supplementary Figures S9G, 9H). These findings were unexpected, since ECM-associated genes, including those related to *Mmps*, are destabilized in muscles from *mdx* mice and DMD patients, compared to healthy muscles (Kumar et al., 2010; von Moers et al., 2005). We next examined MMP-10 protein expression in FACS-sorted cells isolated from *mdx* mice, finding that 25% of the quiescent satellite cells and 80% of the FAPs expressed MMP-10, with the protein accumulating preferentially in FAPs than in satellite cells (Figure 8D). Interestingly, comparisons between cells from *mdx* and wild type mice pointed out that dystrophic condition reduced the percentage of satellite cells expressing MMP-10, without altering the number of MMP-10 positive FAPs (Supplementary Figures S9I, 9J). Similarly, dystrophic condition decreased MMP-10 accumulation in quiescent satellite cells but not in FAPs (Supplementary Figures S9I, 9K). As well as in wild type mice, Lin^−^ cells from the *mdx* mice did not express the protease either at the transcriptional and protein level (Figure 8D; Supplementary Figure S9I).

We next assessed *Mmp10* gene levels in satellite cells, FAPs and Lin^−^ cells from *mdx* mice upon injury insult, finding *Mmp10* transcripts in all cell subtypes. Protease gene expression was highly upregulated in FAPs and Lin^−^ cells, compared to levels found in activated satellite cells (Figure 8E). Interestingly, comparisons between cells from *mdx* and wild type injured mice showed that dystrophic condition reduced *Mmp10* gene levels in activated satellite cells (Supplementary Figures S9L, 9M), but induced gene upregulation in FAPs and Lin^−^ cells (Supplementary Figure S9L). According to these findings, myoblasts from healthy people and DMD patients expressed the protease both at the transcriptional (Figure 8F) and protein (Figures 8G, H) level, but dystrophic condition decreased *Mmp10* expression levels (Figures 8F, I).

Taken together, these findings show that dystrophic condition increases MMP-10 accumulation in muscle but specifically reduces its expression in the satellite cells and myoblasts. Furthermore, our data reinforce the idea that the transcriptional machinery of MMP-10 is induced under replicative pressure, with satellite cells and other muscle-resident cells contributing to muscle providing needs.

## Discussion

We show here that loss of MMP-10 in young mice predisposes satellite cells to acquire features of premature aging, which precipitate a cellular senescence response under proliferative pressure and a decline in functional repair after consecutive injuries.

In the absence of MMP-10, there are profound alterations in the composition of the muscle and the niche ECM. This explains why satellite cells from young MMP-10 deficient mice function as aged cells. Much of the damage to the satellite cells is the consequence of the progressive deterioration of the composition of the niche ECM (Cohn et al., 2002; Bentzinger et al., 2013; Urciuolo et al., 2013; Riederer et al., 2015). The ECM facilitates satellite cell adhesion to the niche via binding to cell surface receptors, allowing cells to sense mechanical signals from the ECM and respond in an appropriate manner (Glaser et al., 2016; Tierney et al., 2016). Consequently, reduction of ECM components or abnormalities in cell receptors (Lukjanenko et al., 2016; Rozo et al., 2016) destabilize the satellite cell link to the niche and causes aging (Liu et al., 2013; Bernet et al., 2014; Price et al., 2014; Sousa-Victor et al., 2014). Therefore, we suggest that loss of MMP-10 in young mice decreases the abundance and integrity of the preferred binding structures for muscle progenitors (e.g., fibronectin, laminin, collagen IV and *β*-dystroglican), disrupting the cell-ECM interaction and contributing to the development of cell aging-associated phenotypic abnormalities. However, MMP-10 loss might also affect integrin activity since integrin receptors are involved in the outside-in signaling initiated by MMP remodeling of the ECM (Gutiérrez‐Fernández et al., 2015). Furthermore, lack of MMP-10 activity would impede the conversion of structural molecules to signaling molecules by releasing small bioactive peptides and growth factors stored within the ECM (Sternlicht and Werb, 2003), which may be necessary to maintain a young (appropriate) microenvironment or to signal to the cells through integrin receptors (Hynes, 2002; Kim et al., 2009; Zeltz and Gullberg, 2016). MMP-10 loss can induce Nox2 (Navarro-Oviedo et al., 2019), which is necessary for reactive oxygen species (ROS) production (Lambeth, 2004). As elimination of excess ROS deflects damage accumulation in aged satellite cells (García-Prat et al., 2016), MMP-10 deficiency would increase oxidative stress, inducing cellular damage and aging. Consistent with this, the activity of Akt, a PI3K pathway downstream protein that can be regulated by MMP-10 (Bobadilla et al., 2014), acts against autophagy in response to increases in ROS levels (Kma and Baruah, 2022). This situation may further explain why reduction of MMP-10 in the satellite cells induces damaged DNA accumulation and activates p16/pRb senescence signaling, while addition of the protein has the opposite effect. Interestingly, integrin signalling maintains mitochondrial bio-energetic function by reducing ROS formation (Visavadiya et al., 2016). Thus, defective integrin activity related to an abnormal degrading process of the muscle ECM, caused by loss of MMP-10, might cause the opposite effect and increase ROS production. Lack of MMP-10 might cause satellite cell aging at other different levels, since the function of the MMP members is extremely complex and they may have unexpected effects on the behaviour of the cells. Future work should establish the consequences of MMP-10 deficiency in the satellite cells in the aging context, depicting if this MMP acts as a ligand, protease or signalling molecule.

The similarities observed between young MMP-10 deficient mice and aged wild type mice, both in muscle ECM composition changes and in satellite cell-autonomous processes (Chakkalakal et al., 2012; Bernet et al., 2014; Sousa-Victor et al., 2014; Lukjanenko et al., 2016; Rozo et al., 2016), highlight the potential role of MMP-10 in the satellite cell aging program. Loss of MMP-10 induces aging features to the satellite cells, promoting breaks in quiescence in a subset of cells, according to the idea that aged niches drive satellite cells out of a dormant state (Chakkalakal et al., 2012). However, if loss of MMP-10 led satellite cells to function as aged cells, one would expect the stem cell compartment should be gradually depleted (Chakkalakal et al., 2012; Sousa-Victor et al., 2014). Here we showed that satellite cell numbers not differed between wild type and mutant mice under healthy conditions, but differences arise under pathological insult. Several studies in muscle regeneration following injury indicate that satellite cells from aged mice accumulate stem cell-intrinsic defects before the depletion of satellite cell numbers is evident in geriatric mice (Collins et al., 2007; Sousa-Victor et al., 2014). Thus, we can speculate that the stem cell compartment in adult MMP-10 KO mice acquires intrinsic defects even before a decline in satellite cell numbers is apparent, with satellite cells and myoblasts becoming less efficient at responding to and repairing damage under replicative pressure. Our cell transfer experiments confirm that satellite cells from mutant mice are less efficient than those from wild type animals and point to MMP-10 loss as the cause of the cellular defect. Additionally, we would speculate a possible mechanism whereby once activated, satellite cell-mediated expression of MMP-10 initiates a cascade of events in the niche ECM, necessary for satellite cell fate decisions. Consistent with this, *Mmp10* transcripts are found in activated satellite cells, increased in myoblasts and declined in myotubes. Upon activation, satellite cells actively remodel the ECM within their niche by producing MMPs and synthesizing ECM proteins (Lukjanenko et al., 2016; Rayagiri et al., 2018), guiding cell expansion, self-renewal or commitment through symmetrical or asymmetrical cell divisions (Feige et al., 2018). Efficient muscle regeneration and homeostasis depends on these instructive signals from the ECM (Webster et al., 2016) and our data suggest that MMP-10 loss, through a dysfunctional proteolytic remodeling of the ECM, modifies these cellular guides. Whether these effects are due to a reduced ability to maintain quiescence, or whether MMP-10 has a direct role following activation and proliferation, remains to be determined. What is clear is that the niche ECM involves both autoregulatory matrix that is secreted by satellite cells into their own microenvironment and molecules produced by supportive accessory cell types, which also participate in the ECM remodeling process (Fuchs and Blau, 2020; Schüler et al., 2022). Here we showed that FAPs express MMP-10 and that expression levels increase in the context of injury, as well as in other resident muscle cells. Inflammatory cells, including eosinophils and macrophages, are recruited to the site of damage and guide the expansion and clearance of resident FAPs in a delicate balance to preserve the stem cell niche (Biferali et al., 2019; Saito and Chikenji, 2021). A shift in this temporally coordinated response can lead to satellite cell dysfunction and defective repair, and it could further precipitate aging (Biferali et al., 2019; Wang et al., 2019; Saito and Chikenji, 2021). Here we found that eosinophils and macrophages are susceptible to age-related DNA damage but also to damage caused by MMP-10 loss, according to the implication of MMP-10 in inflammatory processes (McMahan et al., 2016). This suggest the contribution of inflammatory cells and FAPs to the muscle phenotype observed in the mutant mice. Nonetheless, future studies will have to address the spatiotemporal contribution of individual MMP-10-secreting cell populations to the satellite cell niche.

It is worth noting that MMP-10 accumulates in muscles from old mice, suggesting that aging increases MMP-10 in the whole muscle but induces a decline in the satellite cells. However, our study has a limitation that there is no evidence of MMP-10 diminution in the satellite cells from aged mice. As we detected MMP-10 protein by immunostaining the muscle tissue sections, we were unable to distinguish between the pro-enzyme and the activated protease. However, given the high increase in *Timp1* expression in old muscles, it is tempting to speculate that although MMP-10 protein levels increase, its activity declines through a compensatory mechanism, since TIMP-1 can bind to and inhibit MMP-10. An alternative means of regulating MMP-10 is via their own proteolytic inactivation. However, relatively little is known about this specific process. Nevertheless, it is clear that some cleavages inactivate MMPs, whereas others can generate truncated enzymes that lose their ability to cleave some substrates but retain their ability to cleave others (Nagase et al., 2006; Fanjul-Fernández et al., 2010; Laronha and Caldeira, 2020). These alternative mechanisms to regulate MMP-10 remain to be tested. Additionally, the reasons behind discrepancies between transcriptional and protein expression levels of MMP-10, previously described by others (ZhanG et al., 2007; Zhang et al., 2009), are not known. Protein and gene expression divergence is likely due to either rapid degradation of transcripts specific to those proteins, post-transcriptional control mechanisms, protein translation rates and turnover of proteins (Yi et al., 2008). The dynamic remodeling process of the niche ECM under physiological conditions may require small continuous changes instead of big sudden ones, with the transcriptional machinery active under injury demands, according to previous data (Lukjanenko et al., 2016).

Satellite cells in dystrophic mice, abnormally active by continuous muscle regeneration, increase cellular damage, contributing to cell depletion, tissue degeneration and muscular dysfunction (Zhang et al., 1979; Sacco et al., 2010). Similarly, muscle cells in DMD patients have a diminished repair response as a consequence of profound cellular stress (Blau et al., 1983; Webster and Blau, 1990) and several evidences show senescence involvement in disease progression (Sacco et al., 2010; Aguennouz et al., 2011; Luca Vita et al., 2020), which directly affects the satellite cells (Sugihara et al., 2020). We showed here that MMP-10 treatment protects dystrophic satellite cells against damage accumulation, while preserving muscles from continue damage. Our data suggest that the presence of MMP-10 modulates the muscle ECM, decelerating the muscular turnover rate and suppressing excess muscle regeneration. This will enable cells to spend more time in a quiescent state. This situation further explains why loss of MMP-10 in the *mdx* mice profoundly deteriorates the dystrophic phenotype (Bobadilla et al., 2014). Membrane fragility due to dystrophin deficiency causes intracellular calcium dysregulation, leading to mitochondrial dysfunction and ROS production (Sebori et al., 2018). Thus, as discussed above, we could speculate that MMP-10 administration in the *mdx* mice will reduce ROS levels directly or through its proteolytic function in the ECM. Consistent with this, exogenous MMP-10 decreased damaged DNA accumulation in myoblasts from dystrophic mice and patients. Our study suggests that MMP-10 would be a promising therapy for DMD. However, there are some intriguing results in apparently disagreement with the beneficial effect of MMP-10 in the *mdx* mice that should be clarified. Although compensatory mechanisms or inactivation/proteolytic processes associated with MMP-10 activity may explain protease accumulation in dystrophic muscles or the divergence of *Mmp10* expression levels in resident dystrophic muscle cells under resting or damage situations, these remain to be tested.

## Data Availability

The raw data supporting the conclusion of this article will be made available by the authors, without undue reservation.
