# Supplementary figures and images for: Loss of the matrix metalloproteinase-10 causes premature features of aging in satellite cells
Source: Front Cell Dev Biol. 2023 May 9;11:1128534. doi: 10.3389/fcell.2023.1128534 (PMC10203875; doi:10.3389/fcell.2023.1128534)

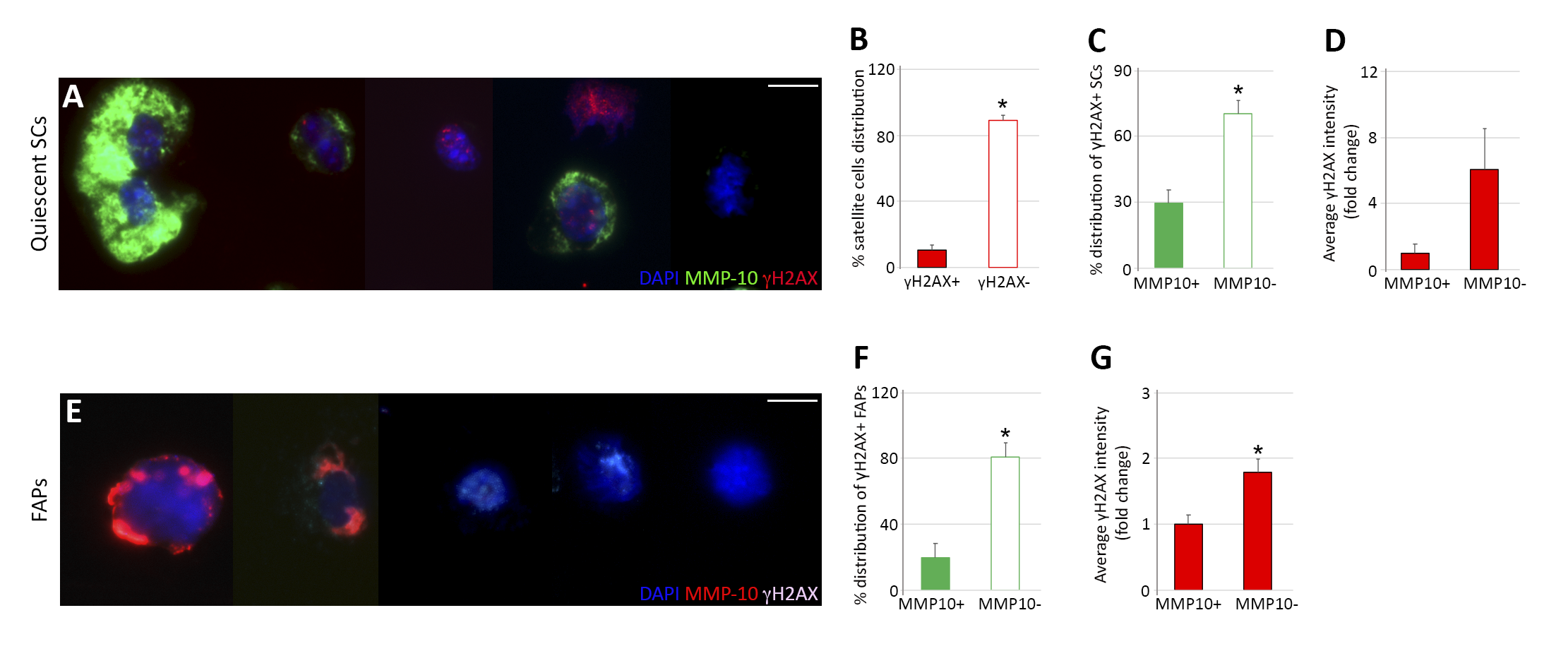

Supplement: Supplementary file 1 [file Image6.TIF]

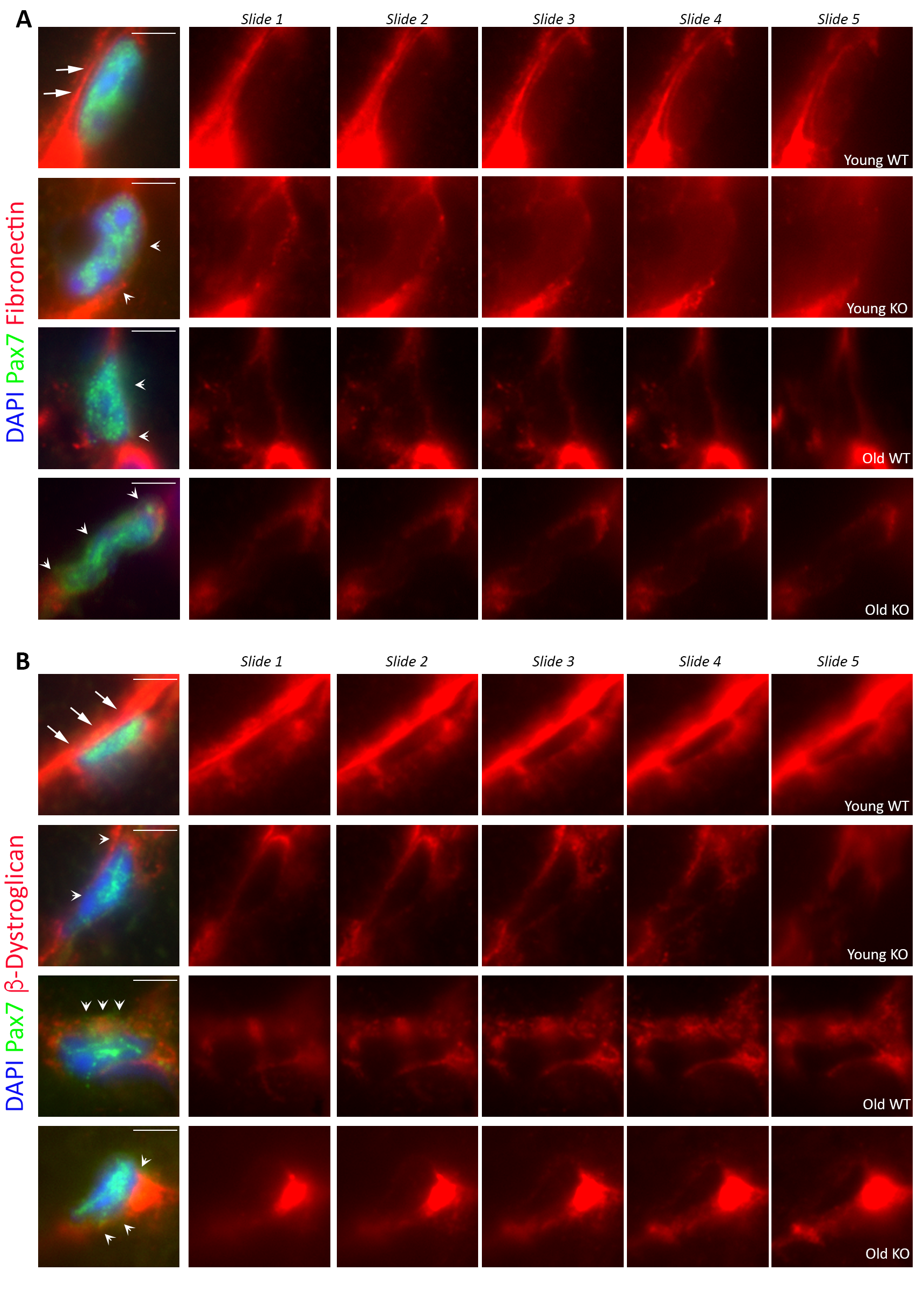

Supplement: Supplementary file 2 [file Image3.TIF]

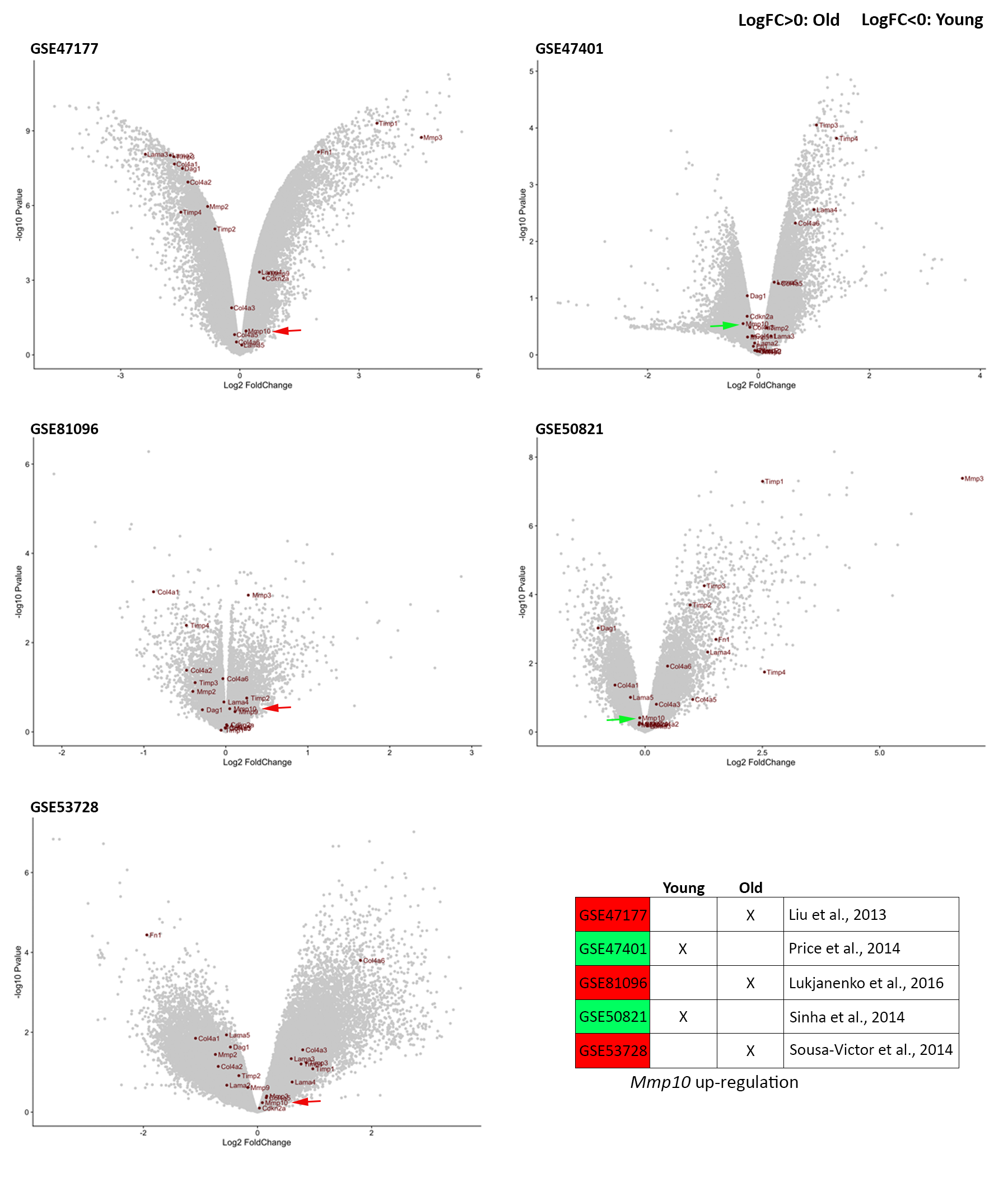

Supplement: Supplementary file 3 [file Image4.TIF]

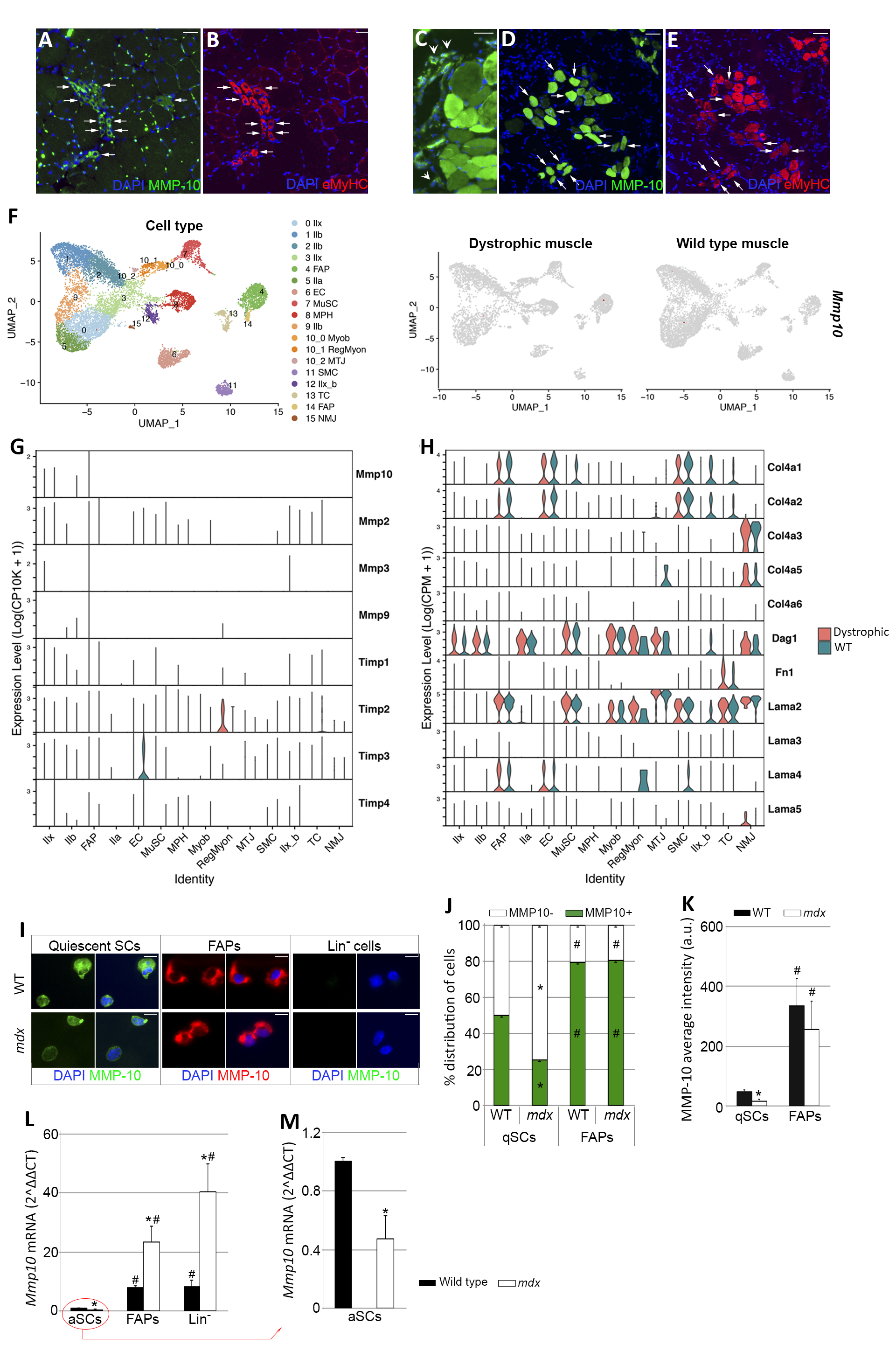

Supplement: Supplementary file 4 [file Image9.TIF]

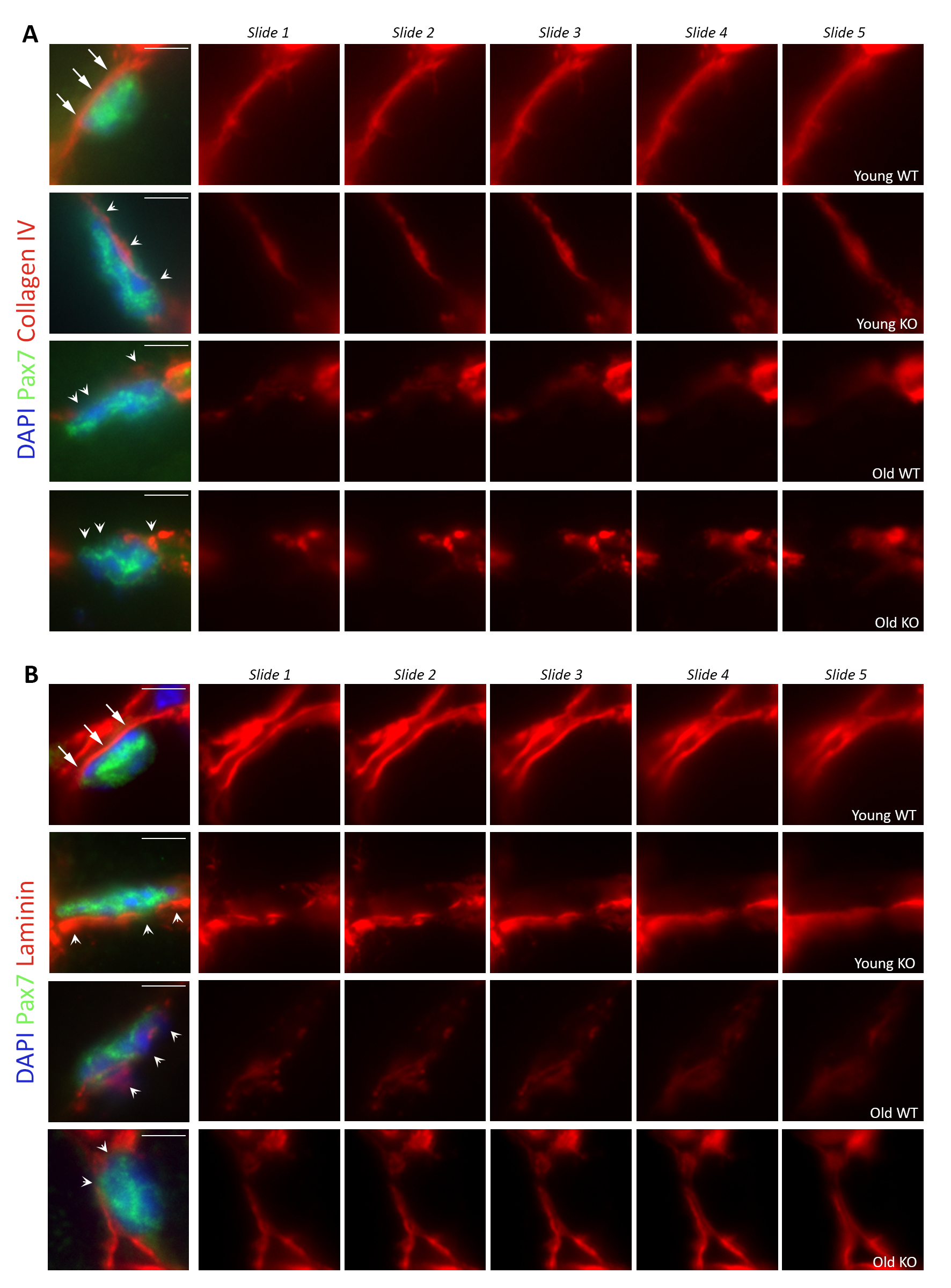

Supplement: Supplementary file 5 [file Image2.TIF]

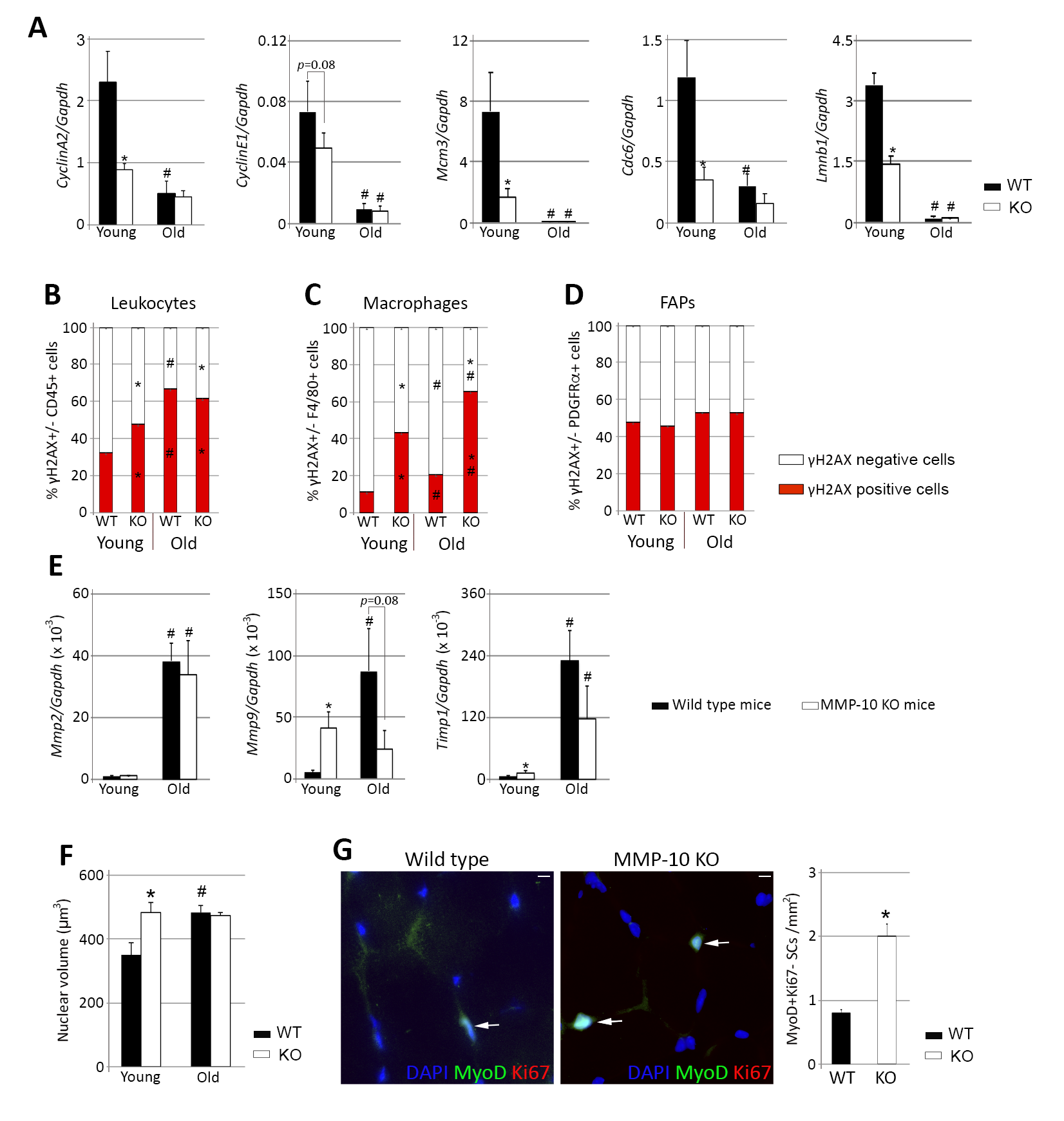

Supplement: Supplementary file 6 [file Image1.TIF]

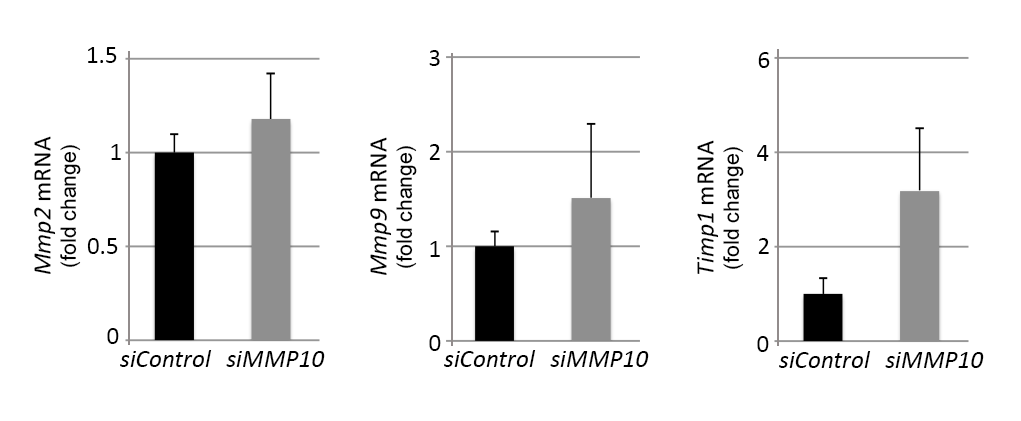

Supplement: Supplementary file 7 [file Image7.TIF]

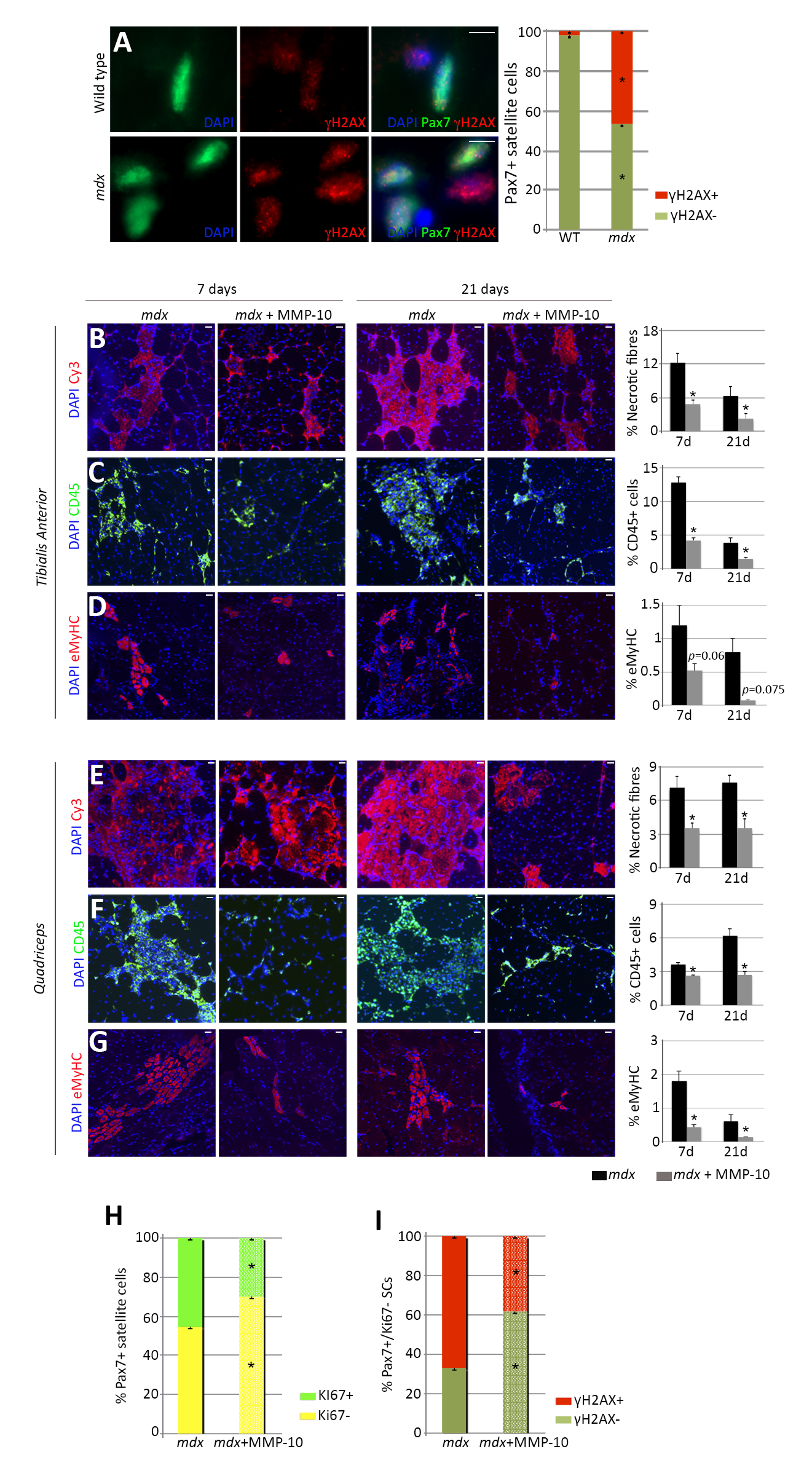

Supplement: Supplementary file 9 [file Image8.TIF]

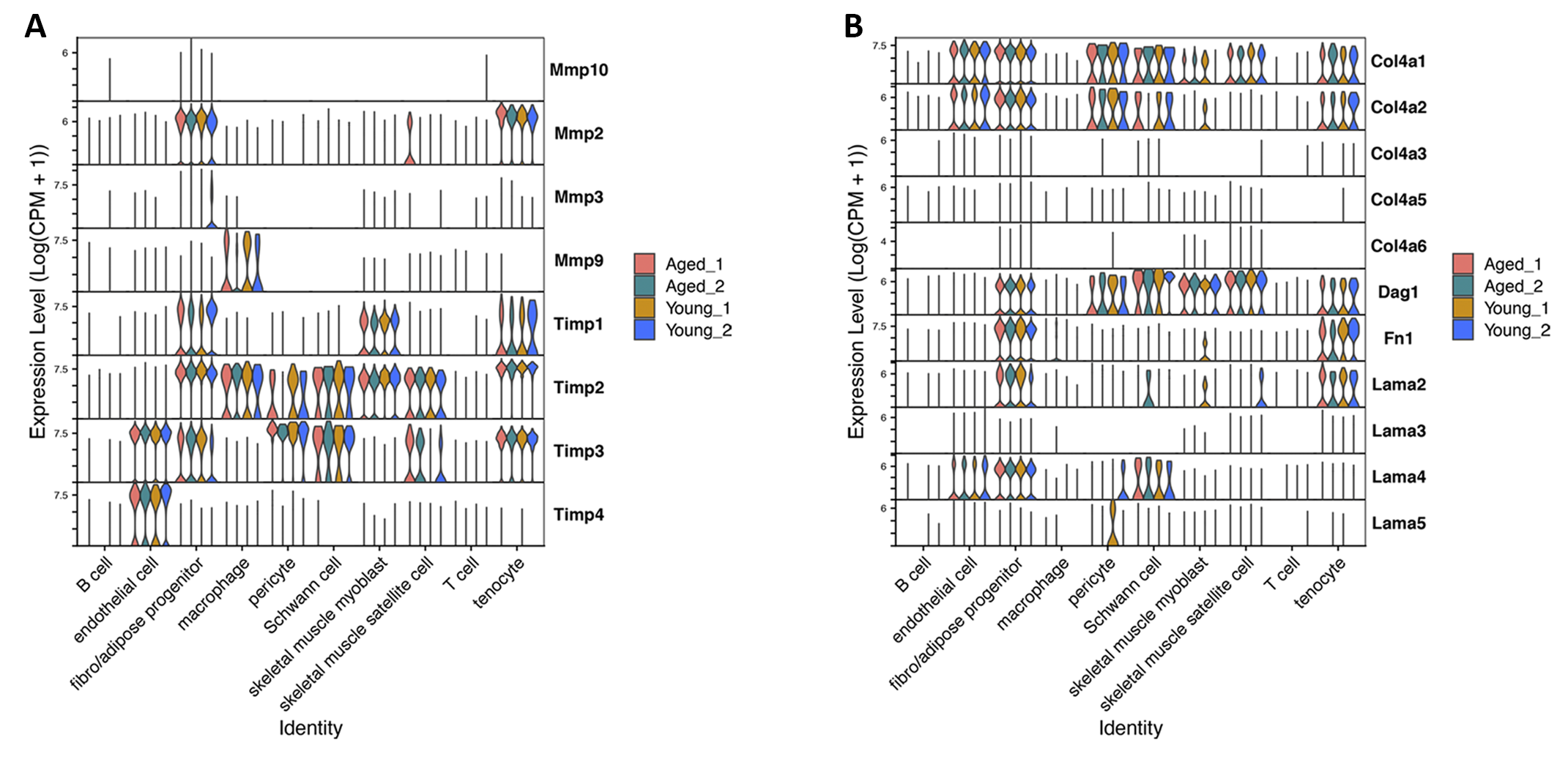

Supplement: Supplementary file 10 [file Image5.TIF]
