# Supplementary material for: Loss of the matrix metalloproteinase-10 causes premature features of aging in satellite cells
Source: Front Cell Dev Biol. 2023 May 9;11:1128534. doi: 10.3389/fcell.2023.1128534 (PMC10203875; doi:10.3389/fcell.2023.1128534)
Supplement: Supplementary file 8 [file DataSheet1.PDF]

Supplementary Material

Loss of the matrix metalloproteinase-10 causes premature features of aging in satellite cells

First Author: Miriam Bobadilla

*** Correspondence:** Ana Pérez Ruiz and Felipe Prósper; aperu@unav.es, fprosper@unav.es

Supplementary Figures

Supplementary Figure 1: Quantification of mRNA expression of pRb-downstream genes (A) in the TA muscles of wild type and MMP-10 KO mice at 2 and 18 months of age, related *Gapdh*. Measurements were related to those from young wild type mice and expressed as a fold change. Quantification of the percentage of leukocytes (B), macrophages (C) and FAPs (D) with damaged DNA. Graphs in (E) show gene expression levels of *Mmp2*, *Mmp9* and *Timp1* in muscles, standardized to *Gapdh*. Nuclear volume size of the satellite cells from wild type and mutant mice at 2 and 18 months of age (F). Representative TA muscle tissue sections from young wild type and mutant mice co-immunostained for MyoD and Ki67 and quantification of $MyoD^{+}Ki67^{-}$ committed satellite cells (G). DAPI was used to identify all nuclei. Scale bar: 40 µm. Measurements are expressed as the mean ± SEM of at least three biological replicates. * designates significance between wild type and mutant mice at the same age, while $^{\#}$ defines significance between young and old mice of same strain where $^{*\#}p<0.05$. KO, MMP-10 knockout; WT, wild type; FAPS, fibro-adipogenic progenitors; SCs, satellite cells.

Supplementary Figure 2: Representative images of muscle tissue sections of wild type and KO mice at 2 and 18 months of age co-immunostained for Pax7 and collagen IV (A) and laminin (B). Images were captured at different Zs (slices) at the same exposure time and equally modified to highlight protein disorganization in the niche ECM. Arrows identified the continuity of the ECM proteins in the $Pax7^{+}$ satellite cells from young wild type mice, while arrowheads highlight an abnormal ECM, with disruptions or rare accumulation of the proteins. DAPI was used to identify all nuclei. Scale bar: 5 µm. Images were equally modified to highlight ECM abnormalities on the satellite cell niche.

Supplementary Figure 3: Representative images of muscle tissue sections of wild type and KO mice at 2 and 18 months of age co-immunostained for Pax7 and fibronectin (A) and β-Dystroglican (B). Images were captured at different Zs (slices) to show protein disorganization in the niche ECM. Arrows identified the continuity of the ECM proteins in the $Pax7^{+}$ satellite cells from young wild type mice, while arrowheads highlight an abnormal ECM, with disruptions or rare accumulation of the proteins. DAPI was used to identify all nuclei. Scale bar: 5 µm. Images were equally modified to highlight ECM abnormalities on the satellite cell niche.

Supplementary Figure 4: *Mmp10* transcript levels associated with published microarrays performed in quiescent satellite cells sorted from young and old wild type mice. Arrows localize *Mmp10* transcripts, which were up-regulated in quiescent satellite cells from young (green) or old (red) mice. Table shows *Mmp10* gene up-regulation in satellite cells from young or old mice, depending on dataset analyzed.

Supplementary Figure 5: Violin plots showing single cell log-normalized expression of MMPs (A) and ECM-related genes (B) for each cell type populations within the skeletal muscles from young and aged mice (Kimmel et al., 2021), splitting by sample of origin.

Supplementary Figure 6: Representative images of FACS-sorted quiescent satellite cells (A) and FAPs (E) co-immunostained for MMP-10 and γH2AX. DAPI identifies all nuclei. Scale bar: 20 µm. Graph B shows the percentage of satellite cells with or without γH2AX marks. Graphs in C and F show the percentage of total γH2AX positive satellite cells and FAPs expressing or not MMP-10. Bars in D and G indicate damaged DNA accumulation in MMP-10 negative satellite cells or FAPs, related to that quantified in MMP-10 positive cells, which was considered 1. Values are expressed as the mean ± SEM of three biological replicates and * designates statistically significant differences (p<0.05). FAPS, fibro-adipogenic progenitors; SCs, satellite cells.

Supplementary Figure 7: Bars show gene expression levels of *Mmp2*, *Mmp9* and *Timp1*, related to *Gapdh*. Measurements in *siMMP-10* treated cells were expressed as a fold change compared to *siControl* cells and expressed as the mean ± SEM of at least three biological replicates. * designates p<0.05 between siMMP-10 treated cells comparing to control cells.

Supplementary Figure 8: Representative images of TA muscles of wild type and *mdx* mice (scale bar: 40 µm) co-immunostained for Pax7 and γH2AX, and percentage of $Pax7^+$ satellite cells accumulating $γH2AX^+$ foci (A). Representative images of TA and quadriceps from control and MMP-10 treated *mdx* mice (scale bar: 20 µm) immunostained for Cy3 (B and E), CD45 (C and F) and eMyHC (D and G) 7 and 21 days after MMP-10 administration. DAPI was used to identify all nuclei. Percentage of $Pax7^+Ki67^-$ and $Pax7^+Ki67^+$ satellite cells (H) and percentage of $Pax7^+Ki67^-$ quiescent satellite cells (I) in muscles from treated and control mice with γH2AX positive foci 21 days after MMP-10 delivery. Data are shown as the mean ± SEM of at least three animals per condition. * identifies significance between control and MMP-10 treated groups where p<0.05. SCs, satellite cells.

Supplementary Figure 9: Representative images of 200 µm^2 consecutive muscle tissues from *mdx* mice (A-B) and DMD patients (C-E) immunostained for MMP-10 (A, C-D) and eMyHC (B, E). Arrowheads in C point out MMP-10 positive monuclear cells. Arrows in A-B and D-E indicate double positive immunostaining. Exposition time to capture image C was higher than that used to capture image D. UMAP representations of cell type populations within the skeletal muscles from wild type and dystrophic mice (Chemello et al., 2020), splitting by sample of origin, where clusters with the same cell type annotation are considered together (F). UMAPs represent the normalized gene expression of *Mmp10* for each sample separately. Violin plots showing single cell log-normalized expression of MMPs (G) and ECM-related genes (H) for each cell type populations within the skeletal muscles from healthy and dystrophic mice, splitting by sample of origin. Representative images of quiescent satellite cells, FAPs and Lin^- cells (scale bar: 20 µm), isolated from wild type and *mdx* mice immunostained for MMP-10 (I), percentage of positive cells for MMP-10 (J) and quantification of MMP-10 average intensity (K). Expression of *Mmp10* mRNA levels in FACS-sorted activated satellite cells, FAPs and Lin^- cells isolated from muscles of wild type and *mdx* mice three days after notexin injection (L). Graph in M amplifies data related to activated satellite cells showed in L. Data are shown as the mean ± SEM of at least three animals per condition. * and # designate significance (p<0.05) between wild type and *mdx* cells from same subtype or between cell subpopulations from same strain, respectively. a.u., arbitrary units; qSCs, quiescent satellite cells; aSCs, activated satellite cells; SCs, satellite cells; FAPs, fibro-adipogenic progenitors; WT, wild type; UMAP, uniform manifold approximation and projection;

IIb, type IIb myonuclei; IIx, type IIx myonuclei; IIa, type IIa myonuclei; IIx_b, type IIx_b myonuclei EC, endothelial cell; MuSC, muscle satellite cells; MPH, macrophages; Myob, myoblasts; RegMyon, regenerative myonuclei; MTJ, myotendinous junction myonuclei; SMS, smooth muscle cells; TC, tenocytes; NMJ, neuromuscular junction myonuclei.
